# Predicting marine habitat for marbled murrelets during breeding and nonbreeding seasons in the Salish Sea, British Columbia, Canada

**DOI:** 10.1371/journal.pone.0316946

**Published:** 2025-01-16

**Authors:** Sonya A. Pastran, Patrick D. O’Hara, Caroline H. Fox, Mark C. Drever, Ross Vennesland, Douglas F. Bertram

**Affiliations:** 1 Wildlife Research Division, Institute of Ocean Sciences, Environment and Climate Change Canada, Integrated Marine Spatial Ecology Lab, Sidney, British Columbia, Canada; 2 Institute of Ocean Sciences, Environment and Climate Change Canada, Canadian Wildlife Service, Integrated Marine Spatial Ecology Lab, Sidney, British Columbia, Canada; 3 Department of Geography, University of Victoria, Victoria, British Columbia, Canada; 4 Environment and Climate Change Canada, Canadian Wildlife Service, Nanaimo, British Columba, Canada; 5 Wildlife Research Division, Environment and Climate Change Canada, Pacific Wildlife Research Centre, Delta, British Columbia, Canada; 6 Environment and Climate Change Canada, Canadian Wildlife Service, Pacific Wildlife Research Centre, Delta, British Columbia, Canada; Auburn University, UNITED STATES OF AMERICA

## Abstract

The marbled murrelet (*Brachyramphus marmoratus*) is a small seabird inhabiting coastal regions along the Pacific coast of North America, and nests in old-growth forests usually within 80 km from shore. The Canadian population of marbled murrelets is listed as Threatened under the federal *Species at Risk Act*. To investigate the species’ marine distribution, we conducted analyses of the occurrence of marbled murrelets at-sea between 2000 and 2022, utilizing at-sea and marine shoreline surveys in the Canadian portion of the Salish Sea. The data were divided into breeding season (April to August) and non-breeding season (September to March) to examine the relationship between environmental covariates and the species’ distribution. We considered terrestrial covariates related to potential nesting habitat, as well as marine covariates related to Pacific sand lance (*Ammodytes personatus*) quality habitat, slope, depth, streams, tidal currents, shorelines and climate indices (NPGO). We compared marine distribution between breeding and nonbreeding seasons and predicted variations in covariate relationships. Our study focuses on identifying averaged relationships and key spatial areas to gauge habitat quality at a landscape scale. Using a Generalized Additive Modelling approach, we found that both marine and terrestrial covariates contributed to predicting murrelet distribution during both seasons. Notably, Pacific sand lance habitat played a significant role in both the breeding and nonbreeding season, while the overall amount of nesting habitat within an 80 km radius influenced occurrence probability in the nonbreeding season. Our analysis accurately predicted distribution patterns at a resolution of 3 x 3 km with an AUC of 0.89 and AUPRC of 0.52 for the breeding season, and AUC of 0.88 and a AUPRC of 0.28 for non-breeding season. Overall, our study highlights both terrestrial and marine drivers that influence the marine distribution of this threatened species and informs Canadian conservation efforts.

## Introduction

Predicting habitat is fundamental for effective conservation planning as it allows for the identification of areas where species are most likely to persist, and contributes knowledge relating to where they are most at risk. Habitat quality is defined as the ability of the environment to provide conditions appropriate for survival, reproduction, and population persistence [1]. Gauging how environmental factors impact seabird distribution can help to identify potential areas of conflict between human activities and vital habitat for species [2], and provide relevant information for proactive mitigation to biodiversity risks. By quantifying key ecological and environmental factors that influence habitat quality, we can identify areas where human uses may pose a threat to the survival of species that rely on those habitats. For example, habitat quality modeling for seabirds can identify areas where species distribution overlap with human activities, and facilitate assessment of potential impacts of fishing, marine vessel traffic, or nearshore/offshore energy development [3–5].

Marbled murrelets (*Brachyramphus marmoratus*, hereafter, murrelets) are small, elusive seabirds which inhabit the coastal regions along the Pacific coast of North America from Alaska to California. They are unique among seabirds because they typically breed in old-growth forests, on rare occasions over a hundred kilometers from the coast, and their young hatchlings are raised on large branches in the canopy of mature trees [6]. In Canada, murrelets nest typically within 30 km from shore, but nests have been located 50 km or more inland [7]. Based on nests found in Canada and the USA, an average foraging range of 80 km has been estimated [8]. Murrelets travel frequently to their nesting sites from coastal foraging areas, having 24-hr incubation shifts shared by both mates, and later in the season travel to bring food to their single nestling [9]. Consequently, foraging locations during the chick rearing period are influenced by the necessity to stay within areas in proximity to the nesting sites [10,11]. Detailed information on how prey abundance influences the identification of quality marine habitat remains notably limited [12,13].

Murrelets exhibit a distinct feeding behavior, carrying only one fish during their inland flights, emphasizing the need for high quality prey for their young [14]. Pacific sand lance (*Ammodytes personatus)* historically has been found to be a vital prey in the Pacific region, has been shown to influence breeding success for various seabird species [15,16], and is a common foraging species for murrelets [17,18]. As Pacific sand lance lack a swim bladder, they are difficult to track with acoustics [19]. Instead, physical characteristics have been used as proxies of potential sand lance habitat. This modeling strategy, using proxies for forage fish, has had varying degrees of success [12,13,20]. However, a growing body of work now exists, and continues to develop, that more accurately models habitat for Pacific sand lance[21–23]. This advancement can now aid in more precise predictions of habitats for predators which rely on these forage fish.

Marine habitat studies on murrelets have predominantly centered on the species’ marine distribution during the breeding season [8,12,20]. Those investigations revealed variations in the utilization of marine foraging areas by murrelets and their associations with physical marine characteristics across different geographic regions [12,24,25], highlighting the importance of regional habitat studies. While the importance of nesting habitat for murrelets is a common finding, the degree of this relationship varies. Haynes (2010) and Pastran (2021) found strong influences from distance to creeks, rivers, or flyway zones, while Raphael and coauthors emphasized distance to nesting habitat and proximity to shore [12,24,25]. Additionally, sea surface temperature, although not as strong as terrestrial influences, still had some relative influence on murrelet density, but was selected out of the top models in Pastran’s 2021 study. Caution should be taken when comparing the relative influences of variables between these studies, as they differ in the number of covariates considered and modeling techniques used. Nonetheless, these studies highlight important similarities and differences in the drivers of murrelet distributions. Notably, comprehensive peer-reviewed habitat modeling studies within the Canadian portion of the Salish Sea have not yet been completed for both the breeding and nonbreeding seasons. The marine distribution of murrelets is an important research topic in the Canadian portion of the Salish Sea, where their numbers have been declining [26–28]. In Canada, murrelets are listed as a threatened species under the federal *Species at Risk Act*, and a recovery strategy has been developed and updated [7]. Within the Canadian portion of the Salish Sea, summer marine habitat was modelled in a 2016 technical report [29] and was included in the Recovery Strategy [7], while the distribution during the non-breeding season has not been formally quantified.

We presume that murrelets, like other auks, select marine environments which optimize energy expenditure during the breeding season [30], particularly by staying in proximity to abundant and intact nesting habitats [12]. We predict that the availability and connectivity of nesting habitats will positively correlate with murrelet occurrence during the breeding season and, to a lesser extent, during the nonbreeding season. Furthermore, we expect that murrelet distribution is driven by a combination of factors related to prey availability and foraging efficiency [31]. These factors include proximity to shore and streams, characteristics of the marine environment (such as bathymetry, tidal currents, and Pacific sand lance habitat), and broader ocean climate patterns. Murrelet occurrence is expected to be more prominent in environments where optimal conditions exist, particularly in zones closer to shores, within shallower waters, steeper topography, more vigorous tidal currents, and key foraging grounds.

To address these hypotheses, our research is guided by three overarching goals. Firstly, we investigate how the quantity and connectivity of potential nesting habitat influences the marine distribution of murrelets. Next, we explore how the distribution of murrelets correlates with additional environmental variables, using model-derived information to predict spatially significant areas during both breeding and nonbreeding seasons. Finally, the robustness of these model-based predictions is evaluated through cross-validation.

## Methods

### Study area

The Salish Sea is a dynamic mosaic of marine ecosystems, stretching from the northern portion of the Strait of Georgia to the southern portion of Puget Sound in Washington state. It is characterized by a series of deep channels, shallow bays, and rocky islands that create a diverse and often productive environment for marine life [32]. The region is heavily influenced by freshwater inputs from rivers, which can affect the salinity and nutrient content of the water [33]. Additionally, strong tidal currents and upwelling events, influenced by ocean depth and slope features, can cause rapid changes in water temperature and nutrient levels, which can influence marine species’ distribution and abundance [33]. This sea encompasses a total area of 16,925 square kilometers, and has undergone significant changes over the years, both marine and terrestrial. On the marine front, overfishing and pollution have significantly impacted the health and sustainability of the ecosystem [34,35]. Climate change has also greatly affected the region, by warming waters and changing ocean chemistry [36]. On the terrestrial side, the region has experienced significant degradation due to forestry, agriculture, and urbanization [37]. This has resulted in the loss and alteration of habitat for many species (including murrelets) that nest in old-growth forests along the coast.

**Fig 1:**
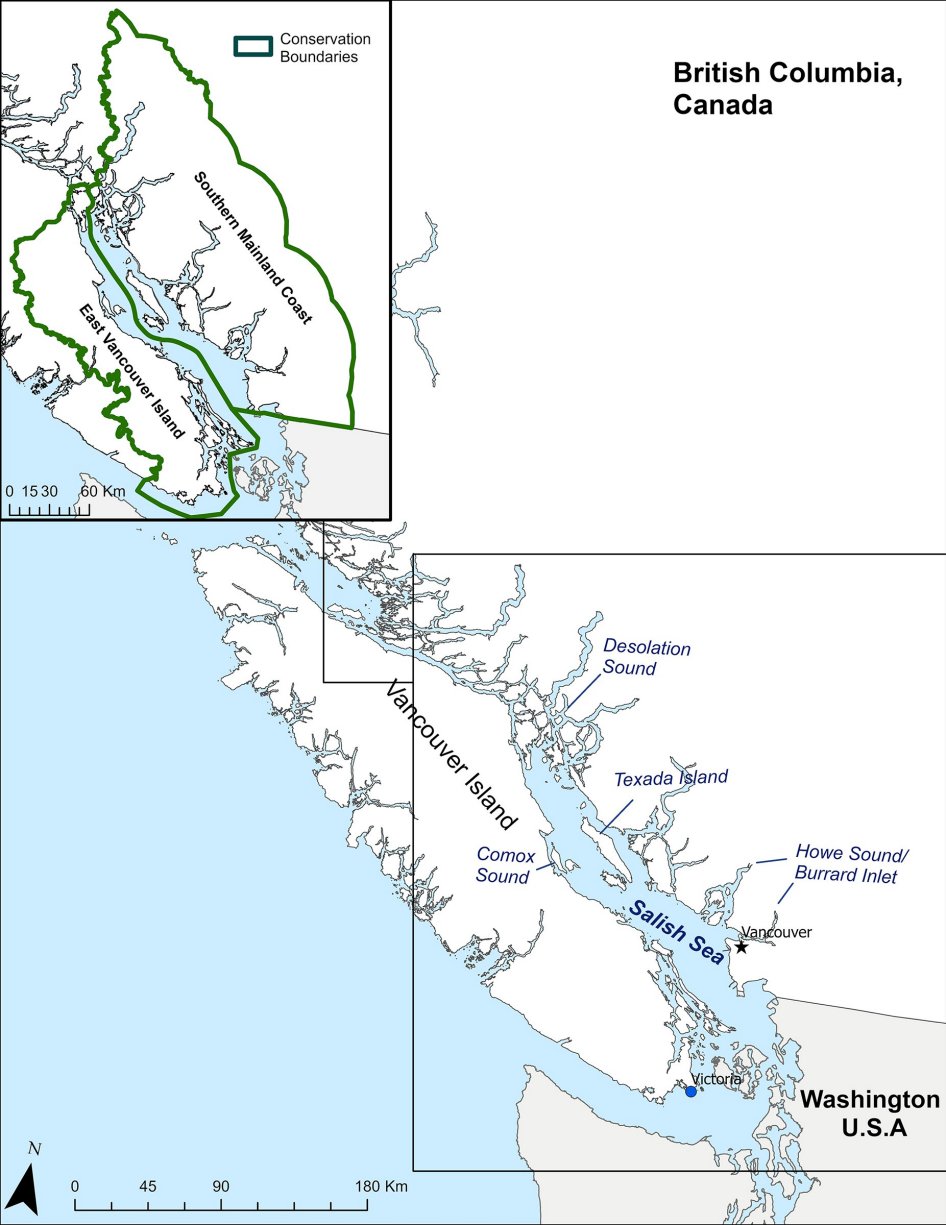
Study area. Map encompassing the general study area and the two marbled murrelet conservation zones used to make study boundaries, East Vancouver Island and Southern Mainland Coast, covering the majority of the Canadian Salish Sea.

Across British Columbia, seven conservation zones have been established for murrelets [38]. We restricted our study to two murrelet conservation zones encompassing the Salish Sea’s Canadian portion: East Vancouver Island and the Southern Mainland Coast (Fig 1). This area includes the Canadian portion of the Salish Sea, an enclosed water body, predominantly bordered by land, featuring estuarine and fjord-like characteristics, along with various human activities. This protected ‘inland sea’ is distinct, setting it apart from the expansiveness of the open Pacific ocean on the outer, exposed coast. The two conservation zones have some contrasting features: The southern mainland zone has a higher human population density [39] and has a higher number of deep fjords that run inland. The eastern Vancouver Island zone experiences cooler water temperatures, influenced by the colder waters of the Pacific Ocean entering through the Strait of Juan de Fuca [39]. In contrast, the mainland zone, particularly in the southern reaches of the strait, tends to have warmer temperatures due to the more limited tidal exchanges [40]. Salinity differences are evident as well, with the eastern Vancouver Island side generally having higher salinity levels compared to the mainland zone. This discrepancy is attributed to the freshwater influx from rivers such as the Fraser River into the Strait of Georgia [41]. On the eastern Vancouver Island zone, human impact is comparatively less pronounced, with more areas characterized by natural landscapes and a lower level of urban development [39].

### Data sources

**Fig 2:**
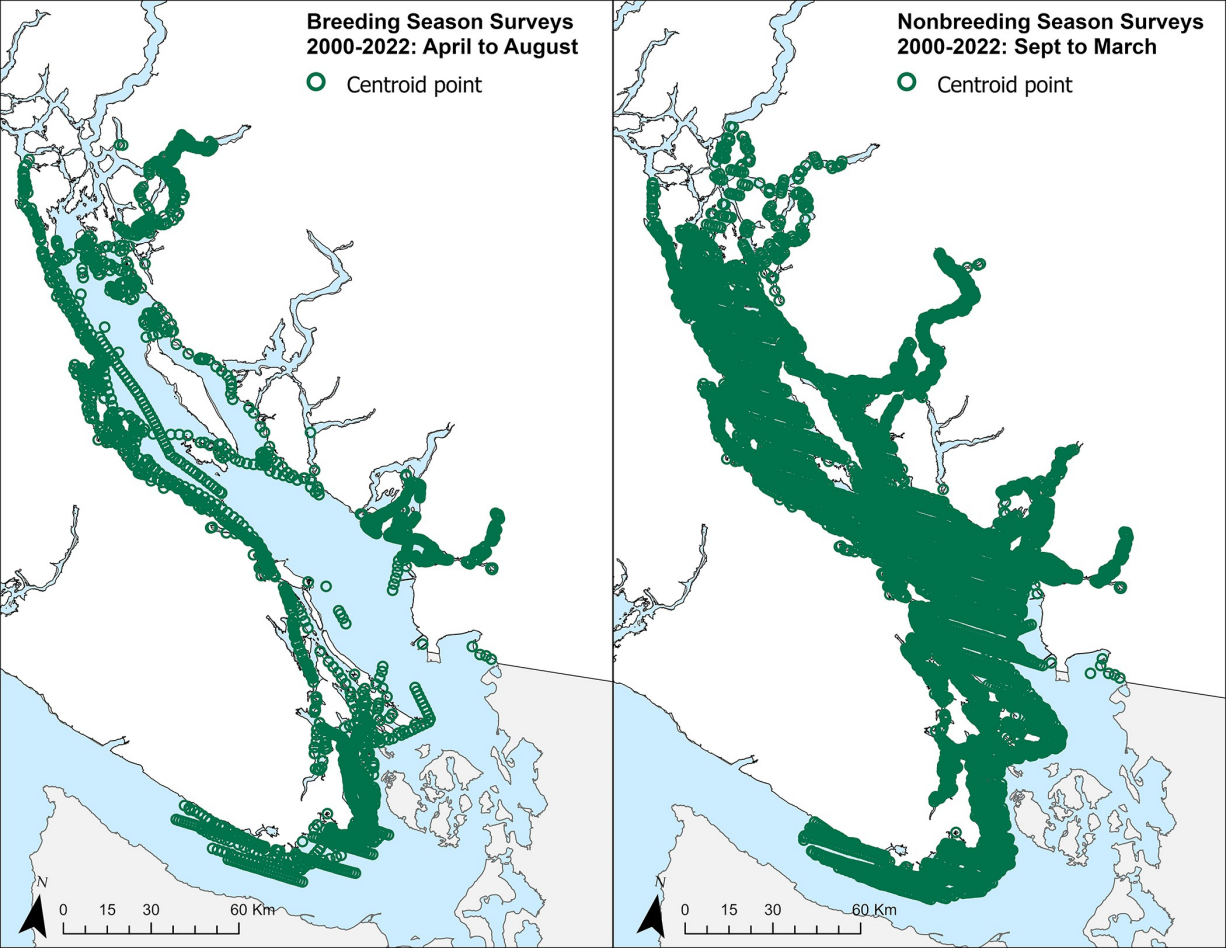
Raw transect centroid points representing survey effort throughout the study region. The left panel represents survey effort for the marbled murrelet breeding season dataset and the right panel represents the survey effort for the nonbreeding season dataset. Both datasets ranged between the years 2000 and 2022, and include marine and shoreline surveys.

We compiled murrelet marine occurrence data, encompassing both presence and absence information, sourced from available records spanning 2000 to 2022 (Fig 2 and S1 Table). To address the spatial and temporal variations in survey efforts within our study region, we combined surveys over time, aiming to construct robust habitat quality models. The decision to exclusively utilize data from 2000 onward was prompted by the need to address challenges arising from the irregular spatial and temporal distribution of survey efforts across different years. Simultaneously, we had to consider the changing environment over time, striking a delicate balance by incorporating enough years to ensure comprehensive spatial coverage of the study region. While the resulting dataset offers broad coverage, it exhibits pronounced spatial variability over time. This spatial variability constrains our ability to explicitly model dynamic changes over time, providing the rationale for prioritizing the development of a habitat quality model primarily grounded in static environmental variables. Moreover, we envision this dataset serving as a valuable resource for informing critical habitat planning. The development of habitat quality models that identify the most important spatial areas on a holistic scale is fundamental to Canadian conservation efforts.

We classified our data collection into two primary types: Shoreline surveys and Marine surveys. Shoreline surveys were conducted from shore and were either run as point counts using a spotting scope, from a single location, or were done as shoreside transects looking out on the water using binoculars. A centroid was assigned to reflect the intermediate distance observers were looking out from shore. Marine surveys were conducted on a variety of vessels, with a set transect width, ranging from 100 to 300 meters on either side. It also included transects initially collected using distance sampling collection techniques [42], which we harmonized by confining the observations to within a 200 m strip transect width. A similar technique was done with the North Pacific Pelagic Seabird Database, which restricts distance sampling data to a 300 m width [43]. We opted for a more conservative restriction that aligned best with the other strip width transect data used for these analyses. We only included surveys for both shoreline and marine that had a maximum transect length of 3 kilometers. Due to the variability of the data collection, contributors, and methodologies, we reduced the counts to presence and absence information, and did not explicitly model detectability as these datasets were not designed for fitting an occupancy model. We recorded the contributors as Survey ID, which we defined as a group of surveys run by the same contributor on the same day.

We incorporated survey type as a covariate in our analyses to address their inherent spatial differences. To explore how these survey types interact with various covariates and influence the probability of murrelet occurrence, we conducted additional analyses. Our exploration allowed us to comprehensively assess the impact of survey type on the relationship between covariates and the likelihood of murrelet presence. Detailed comparisons of predictions, marginal plots, and cross-validation analyses between the two survey types are provided in S1 File of the Supporting Information section. These findings, and their broader implications, are further discussed in the main manuscript.

### Environmental predictors

We selected and developed environmental covariates that have been found to affect the marine distributions of murrelets and other seabirds (Table 1), and built models to test their relevance. Spatial data were projected using the NAD83 BC Albers projection, standard for the area. These covariates included variables related to potential nesting habitat, Pacific sand lance quality habitat, distance to streams, tidal currents, climate patterns (North Pacific Gyre Oscillation), distance to shorelines, inlets, slope, and depth (Table 1). Marine data was extracted using a 3 km radius and relationships to distance features were measured from survey centroid points, to correspond with the maximum transect lengths. Latitude and longitude were included to account for spatial autocorrelation.

**Table 1:** Covariates considered with respect to marbled murrelet distribution for both the breeding and non-breeding season analyses. Survey ID and Year were used as Random effects. Note that not all covariates listed were considered in the final modeling process.

Covariates	Working Name	Habitat	Rational	Definition
Latitude/Longitude	Lat/Lon		Coordinates to account for spatial autocorrelation.	Geographic coordinate system.
Nesting habitat area	NEST_area_	Terrestrial	Proximity to abundant nesting habitat has had positive relationship to occurrence in marine waters in various regions [12,13,24].	Amount of potential nesting habitat available within an 80 km radius from marine point.
Nesting habitat area (distance weighted)	NEST_areaweight_	Terrestrial	Proximity to abundant potential nesting habitat has had positive relationship to occurrence in marine waters in various regions [12,13,24].	Amount of potential nesting habitat available within an 80 km radius from marine point, with area within 30 km of point having highest weight, 30 to 50 km from point intermediate weight and 50 to 80 km lowest weight.
Nesting habitat cohesion	NEST_coh_	Terrestrial	Intact habitat is has been shown to increase the likelihood of nesting [28,42].	The percent connectedness of potential nesting habitat relative to other potential nesting habitat within an 80 km radius from marine point.
Nesting habitat cohesion (distance weighted)	NEST_cohweight_	Terrestrial	Intact habitat is has been shown to increase the likelihood of nesting [28,42].	The percent intactness of potential nesting habitat relative to other potential nesting habitat, distance weighted. Within 30 km of point having highest weight, 30 to 50 km from point intermediate weight and 50 to 80 km lowest weight.
Pacific sand lance habitat	PSL	Marine	A vital prey species to marbled murrelets in various regions [14,17].	Pacific sand lance quality intertidal and subtidal habitat. Measured in percentage.
Distance to streams	STREAM_dist_	Marine	Have been observed to use streams/rivers as flyways to bring food to nests [26] and distance to streams has been documented as a significant covariate in other marine habitat studies [12].	Measured in meters, as the distance from the center of a segment to stream mouth, of stream classes 3 or higher.
Distance to shores	SHORE_dist_	Marine	Occurrences have been positively linked to being close to shore [12,24].	Measured in meters, as the distance from marine point to shore.
Tidal currents	Tidal	Marine	Higher tidal currents correspond with a higher amount of mixing and can influence the productivity of an area [31].	Modelled tidal current value
North Pacific Gyre Oscillation	NPGO	Marine	Seabird occurrence linked to oceanographic climate variability [44].	Average climate index binned by season and year
Depth	Depth	Marine	Affinity to shallower waters in many regions [12,13,24]	Average water depth in meters, within 3 km radius boundary
Slope	Slope	Marine	The slope of the seafloor has been linked to marine bird distribution [4]	Average seafloor slope in degree value within 3 km radius boundary.
Inlets	Inlets	Marine	Murrelets frequently been observed within inlets [26].	Physical representation based on parks Canada sub-oceanographic regions

Spatial layers of quality intertidal and subtidal habitat for Pacific sand lance were obtained from predictive models based on a maxent framework and were combined into a single raster (S1 Fig) with a uniform 50-meter resolution in ArcGIS Pro 3.1.0 [22,23]. The average probability of presence within a 3 km grid radius of observation points was extracted using the raster package in R 4.2.1. Bathymetry data and slope were derived using the Spatial Analyst Slope tool in ArcGIS Pro, with average values within a 3 km radius of survey centroids extracted [45]. Tidal current information, obtained from modeled values by Foreman et al. (2008), used RMS tidal speeds over modeled tidal cycles, with average tidal currents within a 3 km radius extracted [46]. Distances to shorelines and streams (orders 3 and higher from the British Columbia Stream Atlas Network) were determined using the Near tool in ArcGIS Pro [47]. North Pacific Gyre Oscillation (NPGO) data spanning 2000–2022 were downloaded and binned by averaging values by murrelet breeding and nonbreeding seasons and year [44,48]. A murrelet nesting habitat quality layer (S1 Fig), developed in 2010 and updated in 2018, predicts the amount and distribution of potential nesting habitat on the coast of British Columbia and was built to aid in broad-scale habitat assessments [38,49]. Previous research has shown that the amount of available nesting habitat, its cohesion, and distance to quality areas are related to murrelets’ marine distribution and abundance [8,11,13]. To explore these relationships, we calculated four covariates from the potential nesting habitat layer. Using the *landscapemetrics* package in R 4.2.1, we first calculated the total area of potential nesting habitat within an 80 km radius from marine points. We also calculated a cohesion metric within the same radius [50]. For our analysis, cohesion was characterized by the connectedness of potential nesting habitat. Next, we calculated the same two metrics but used inverse distance summed weights equal to 1, to capture how distance played a role in habitat selection. We used a weight of 0.7 for distances from 0 to 30 km from the marine point, a weight of 0.2 for distances from 30 to 50 km, and a weight of 0.1 for distances from 50 to 80 km. These distances were chosen based on decreasing importance to murrelets [7,8]. For the detailed account of data assembly and extraction, please refer to the S2 File.

### Analytical approach

#### Explanatory GAMs

Our goals were to predict where murrelets might occur, based on gauging how underlying environmental covariates relate to the birds’ distribution using marine survey data [51,52]. We used a Generalized Additive Models (GAMs), which are particularly useful in capturing the nonlinear relationships between the response variable and environmental covariates [53,54]. Because of the various survey contributors and survey types, we decided to use the presence/absence information and exclude the count information. We modeled the presence or absence of murrelets with a binomial GAM (logit link function). We assigned survey ID as a random variable to account for variability in detectability resulting from different methodologies and sampling dates. We also added year as a second random variable to account for year to year variation in murrelet presence. The restricted maximum likelihood (REML) was used to limit overfitting while estimating smoothing parameters [54]. All environmental covariates were centered and scaled with standard deviation. Including latitude and longitude as an interactive term in GAM habitat models may account for spatial autocorrelation [53]. However, if the environmental covariates explain enough of the variation, the inclusion of the interacting coordinates is not essential [53]. After constructing the global model, we tested for spatial autocorrelation using a correlogram test, which calculates Moran’s I value over increasing spatial lags. Moran’s I values of zero indicate there is no spatial autocorrelation on the residuals at that given lag [55,56]. We conducted the correlogram test on the raw counts, the model residuals, and the model residuals of a GAM that also included coordinates as interacting predictors. By comparing these plots, we concluded that it was necessary to include the interaction term between latitude and longitude to account for spatial autocorrelation (S2 Fig). The structure of each model is defined by smoothing functions (s()) applied to the various covariates. For latitude and longitude we set k to 100 to capture spatial autocorrelation. For the environmental covariates, we limited k to 5 to assist in covariate interpretability and overfitting. Additionally, an offset term was included, accounting for survey effort (effort in area survey was logged to match the function of the GAM).

#### Model construction

To ensure the robustness of our models, we assessed multicollinearity among the predictor variables using the concurvity function in R. Concurvity measures the extent to which one predictor in a GAM can be approximated by a smooth function of other predictors. High concurvity values indicate potential multicollinearity, which can compromise the stability and interpretability of the model coefficients [57]. We used these values to ensure that no two or more covariates with high correlation (concurvity > 0.75) were included in the same candidate model together [58,59]. We did not assess concurvity between the random variables in the model and the spatial component, represented by the smooth function of latitude and longitude. This decision was based on the assumption that the random effects and spatial terms serve distinct purposes within the model structure, with the random effects accounting for hierarchical data structures and the spatial term capturing underlying spatial autocorrelation that is not captured by other predictor variables. When two covariates were found to be highly correlated, we employed a comparative approach to determine which variable to retain in the subsequent models. Specifically, we ran univariate models for each competing covariate, assessing their individual contributions to the response variable and comparing using the lowest Akaike Information Criterion (AIC). Models with ΔAIC< 2 were considered to have substantial support from the data relative to other candidate models [60].

The covariate that demonstrated a more parsimonious relationship with the response variable in these univariate model comparisons were then included in the subsequent GAMs. Testing for multicollinearity was done separately for the breeding and nonbreeding season datasets. Upon initial examination, we found that depth and slope were highly correlated for both the breeding and nonbreeding season datasets. Notably, the nonbreeding season dataset showcased a pronounced correlation between distance to shore and depth, as well as depth and slope (see S2 Table), likely due to the greater spatial coverage in the nonbreeding season dataset. After AIC assessment of the univariate models using the breeding season dataset, we retained slope over depth. For the nonbreeding season dataset, we retained distance to shore and slope over depth. The candidate models ranked through AIC for the terrestrial covariates were nesting area size (NESTarea), nesting area weight (NESTareaweight), nesting coherence (NESTcoh), and nesting coherence weight (NESTcohweight) (Table 2). For the breeding season dataset, NESTarea and NESTcoh were highly correlated, while for the nonbreeding season dataset, NESTareaweight and NESTcohweight were highly correlated. As was done with the highly correlated marine covariates, to determine which nesting covariate variant best related to murrelets, we ranked four univariate candidate models. Each model incorporated distinct covariates that relate nesting habitat to murrelets in their marine environment.

**Table 2:** Comparison of four univariate candidate generalized additive models (GAMs) predicting marbled murrelet distribution in the Canadian Salish Sea using different potential nesting habitat covariates for the breeding and nonbreeding seasons. The models also include survey effort as an offset and account for random effects of Survey ID and Year.

Nesting Habitat Parameter	AIC_c_	ΔAIC_c_	R^2^ GAM	% Devience Explained
**Breeding season**				
Cohesion index of potential nesting habitat (distance weighted)	1756.30	0.00	0.24	29.4
Area of potential nesting habitat	1758.10	1.80	0.23	30.1
Area of potential nesting habitat (distance weighted)	1785.83	29.53	0.22	28.0
Cohesion index of potential nesting habitat	1792.90	36.60	0.22	28.9
**Nonbreeding season**				
Area of potential nesting habitat	4660.43	0.00	0.11	21.7
Cohesion index of potential nesting habitat (distance weighted)	4705.66	45.23	0.11	20.8
Cohesion index of potential nesting habitat	4761.02	100.59	0.10	20.1
Area of potential nesting habitat (distance weighted)	4774.74	114.31	0.10	19.5

Along with the specified nesting habitat covariate each candidate univariate model included the random effects SurveyID and Year, as well as the survey effort offset term. The AIC results suggested that during the breeding season, the Cohesion Index of potential nesting habitat (Distance Weighted) was the most effective, explaining 29.4% of the variability, while during the nonbreeding season, the model based on the area of potential nesting habitat performed best, accounting for 21.7% of the variability in the response variable. (Table 2).

We evaluated two global models, both fitted using the fast restricted maximum likelihood (fREML) method, with the select option for automatic smoothing parameter selection, also known as a double penalty shrinkage approach [61]. This method applies an extra penalty to a smooth term by penalizing functions in both the null and range space. The double shrinkage technique performs model selection in one step, allowing us to efficiently identify and remove terms that are not significant [61]. Any variables that are eliminated using this method are designated with zero degrees of freedom within the smoothing terms [61]. The first global model was built using data collected during the breeding season:

logit(yi)=β0+s(Longitudei,Latitudei,k=100)+s(NESTcohweighti,k=5)+s(NESTareai,k=5)+s(PSLi,k=5)+s(STREAMdisti,k=5)+s(SHOREdisti,k=5)+s(Slopei,k=5)+s(Tidali,k=5)+s(NPGOi)+Inletsi+SurveyTypei+u(SurveyIDi)+v(Yeari)+offset(log(Efforti))

And the second global model was built using data collected during the nonbreeding season:

logit(yi)=β0+s(Longitudei,Latitudei,k=100)+s(NESTcohweighti,k=5)+s(NESTareai,k=5)+s(NESTcohi,k=5)+s(PSLi,k=5)+s(STREAMdisti,k=5)+s(SHOREdisti,k=5)+s(Slopei,k=5)+s(Tidali,k=5)+s(NPGOi)+Inletsi+SurveyTypei+u(SurveyIDi)+v(Yeari)+offset(log(Efforti))

Where y represents the logit-transformed probability of presence for observation i, β0 is the intercept and u and v represent random effects. The models includes smooth functions s(⋅) and basis function k.

### Model evaluation

We evaluated the performance of the final model and assessed its predictive accuracy using standard 10-fold cross-validation techniques. For training, we fitted models using 70% of randomly selected data, and tested model predictions against the remaining 30% test data, mimicking the real-world scenarios where the model is used for prediction [62,63]. To further evaluate the model’s reliability, we used a 10-fold cross-validation technique to repeatedly split our data into 10 equal parts. This technique helped to reduce the variance in our evaluation results by averaging the performance over 10 different test sets [62].

We reported the amount of variation explained by our model using the percent deviance explained for both model parts. We also assessed the predictive accuracy of our model by using the area under the receiver operating characteristic curve (AUC) as a threshold independence measure [63]. This measure evaluated how well our model distinguished between occupied and unoccupied cells. A high AUC value, such as 0.9, indicates that the model has strong predictive power and can accurately discriminate between the two classes of data. In contrast, an AUC of 0.5 indicates that the model performs no better than random. We also implemented the area under the precision-recall curve (AUPRC), which plots the precision (positive predictive value) against the recall (sensitivity) [64]. Precision is the ratio of correctly predicted positive observations to the total predicted positives and negatives, while recall is the ratio of correctly predicted positive observations to all actual positives. AUPRC ranges from 0 to 1, but since its minimum increases with prevalence there is no established cutoff for identifying adequate models, though higher indices indicate better model prediction.

**Predictions.** In our examination of the GAMs, we assess the impact of each significant covariate on the probability of murrelet occurrence. For this assessment, we calculated the marginal effects for each covariate while holding all other variables constant at their mean values. This approach allowed us to isolate the influence of individual covariates on occurrence probability, providing a clear understanding of how each factor contributes to the likelihood of murrelet presence. We visualized marginal plots using the mgcViz package in R 4.2.1, alongside the precision of corresponding confidence intervals and the recorded p-values to visualize covariates’ contributions. We present only the plots of covariates that demonstrated a statistically significant influence (P < 0.05) in the results section. To help describe covariates relationships to the probability of occurrence, the x-axis range from each marginal plot was divided into two equal halves, representing the first and second 50% quartiles. The absolute differences in predicted probability between the start and end of each half were calculated to quantify the change in occurrence probability across these segments. Subsequently, the percent change in occurrence probability was calculated by dividing the absolute differences by the starting probability for each segment and multiplying by 100. These differences and percent changes were then summarized and reported in the subsequent section.

Our final models, which we fitted using all available data, were used to predict the distribution of murrelets onto a 3 x 3 km grid. Survey effort was assigned a 9 km^2^ to reflect the 3 x 3 km grid size. We refer to the resulting mapped products as the mapped quality marine habitat. We made predictions using the predict.gam function from the mgcv package in R 4.2.1. We modelled and visualized probability of presence for murrelets’ breeding season and nonbreeding season separately. The cartographic work for the final projected prediction maps was done using ArcGIS Pro 2.8.8, with boundaries defined by the following coordinates 50.9960°N at the top, 48.0916°N at the bottom, 125.3022°W on the left, and 122.4452°W on the right.

## Results

Between 2000 and 2022, a total area of 1,683.04 km^2^ was surveyed from April to August for the breeding season. Out of 3,839 observation points, 337 (8.8%) indicated positive observations. During the nonbreeding season (September to March) within the same years, the survey covered an area of 6,673.51 km^2^. Of the 18,001 observation points recorded in the nonbreeding season, 644 (3.6%) showed positive sightings of murrelets.

The GAM predicting probability of presence during the breeding season exhibited a satisfactory fit to the data, explaining 41.6% of the deviance (S3 Table). This level of explained deviance indicates that our model was able to capture a substantial portion of the variation in marbled murrelet presence during the breeding season. The GAM for the non-breeding season explained a lower percentage of deviance (33.4%; S3 Table). We conducted 10-fold cross-validation tests to further assess the predictive performance of both models. The breeding season GAM yielded an area under the receiver operating characteristic (AUC) curve value of 0.89 and an AUPRC value of 0.52, while the non-breeding season GAM generated an AUC value of 0.88 and an AUPRC value of 0.28. The higher AUC value for the breeding season model (0.89) demonstrates a slightly stronger ability to discriminate between high quality and low quality habitats for murrelets compared to the non-breeding season model (0.88), and the precision recall is also almost two times stronger for the breeding season model.

**Fig 3:**
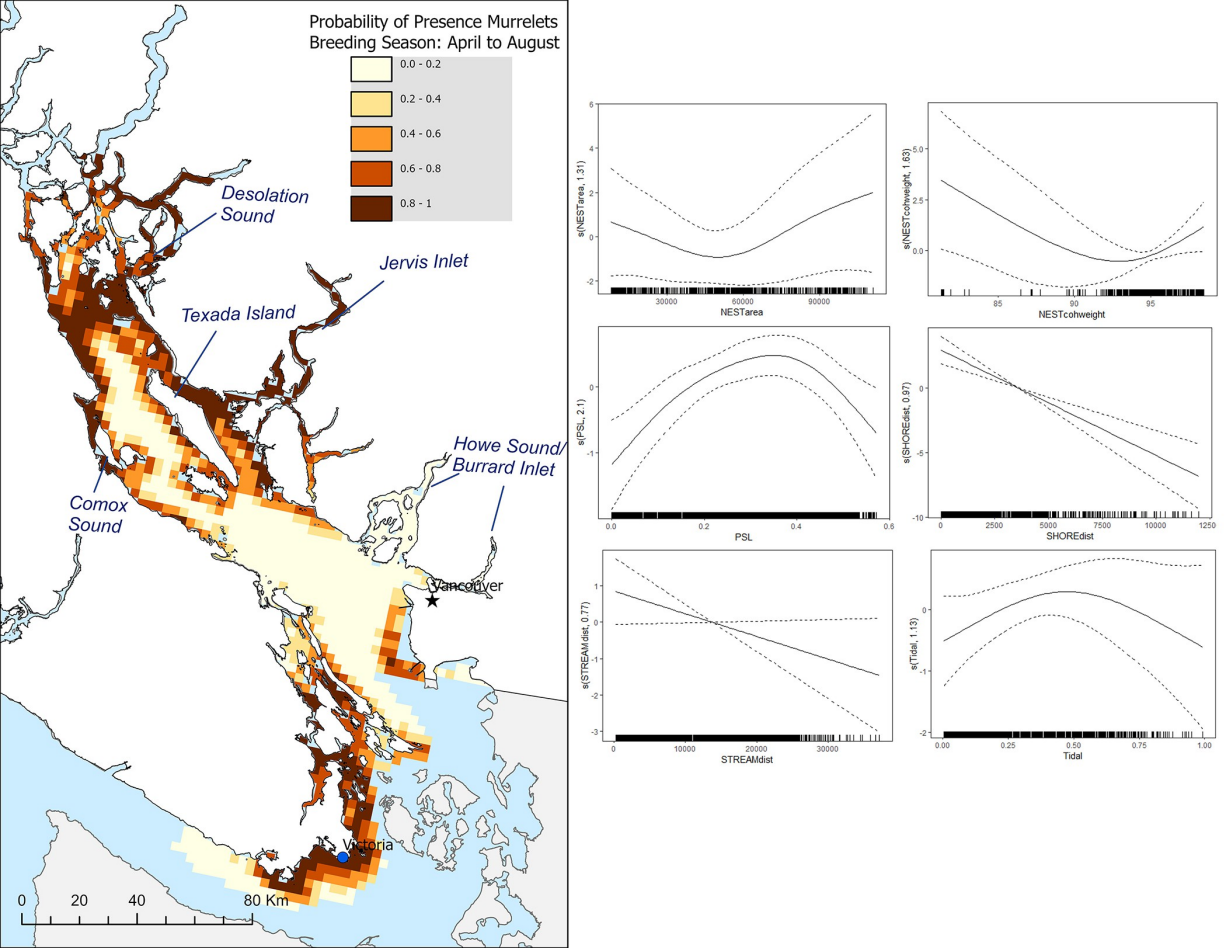
Marine habitat quality map and GAM marginal plots for the breeding season. Predicted probability of marbled murrelet presence during their breeding season (Left), derived from the presence-absence GAM model and projected onto a 3 x 3 km grid and the model terms (Right). For the model terms, estimated smooth functions (solid lines) with confidence intervals (dashed lines) are shown for each explanatory covariate. Tick marks on the x-axis indicate the observed values of the predictor variable on the response variable.

During the breeding season, the marginal effects analyses (Figs 3 and S3) reveal a significant association between the probability of murrelet presence and several key environmental covariates. For NESTarea, a 78.84% decrease in occurrence probability was observed across the lower half of the covariate’s range (4,021.99–50,974.33 m^2^), while the upper half (50,974.33–105,970.65 m^2^) showed an 86.05% increase. This suggests that as NESTarea increases beyond approximately 51,000 m^2^, there is a sharp rise in the probability of murrelet occurrence, indicating that larger nesting areas relate to a higher probability of occurrence. NESTcohweight showed a 90.01% decrease in probability across the lower half of its range (83.76–91.02), followed by a 61.30% increase in the upper half (91.02–98.28). PSL increased the probability of occurrence by 453.15% across the lower half of its range (0.08–0.24) but decreased it by 174.08% in the upper half (0.24–0.55). This indicates that intermediate values of Pacific sand lance habitat quality related to higher probability of occurrence. Tidal currents led to a 217.59% increase in the lower half of its range (-0.02–0.67), with a subsequent 207.30% decrease in the upper half (0.67–1.19). This suggests that moderate tidal currents are optimal for murrelet presence, whereas stronger currents might decrease the probability of occurrence. STREAMdist was associated with a 68.08% decrease in the lower half of its range (2,045.19–23,988.94 m), and a 216.67% decrease in the upper half (23,988.94–38,814.77 m). This finding indicates that murrelets are more likely to occur closer to streams, with the probability sharply decreasing as the distance increases. SHOREdist showed a 99.02% decrease in the lower half of its range (943.04–5,602.4 m) and a 10382.50% decrease in the upper half (5,602.4–8,708.83 m). This suggests murrelet presence significantly declining as the distance from the shore increases. Results suggest that during the breeding season, slope and NPGO have an insignificant influence of murrelet probability of occurrence (S3 Table).

**Fig 4:**
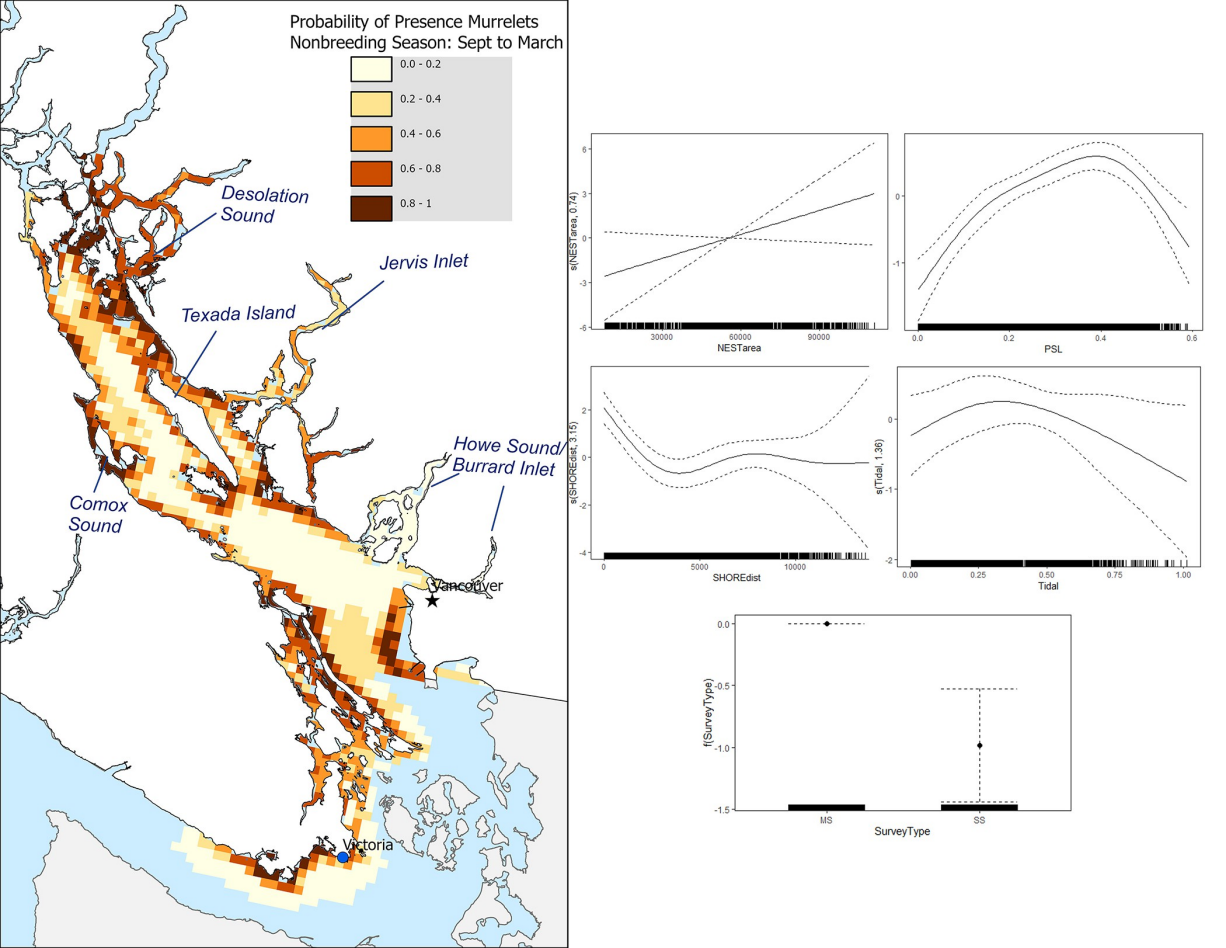
Marine habitat quality map and GAM marginal plots for the nonbreeding season. Predicted probability of marbled murrelet presence during their nonbreeding season (Left), derived from the presence-absence GAM model and projected onto a 3 x 3 km grid and the associated model terms (Right). For the model terms, estimated smooth functions (solid lines) with confidence intervals (dashed lines) are shown for each explanatory covariate. Tick marks on the x-axis indicate the observed values of the predictor variable on the response variable.

In the nonbreeding season, the marginal effects analysis (Figs 4 and S4) indicate a significant and positive relationship between the presence of murrelets and the amount of potential nesting habitat area. NESTarea showed a 1339.16% increase in occurrence probability across the lower half of its range (6,042.87–80,181.37 m^2^), while the upper half (80,181.37–104,894.2 m^2^) displayed a smaller, yet positive, 78.76% increase. This suggests that large nesting areas positively relate to the probability of murrelet occurrence the nonbreeding season. PSL resulted in a 452.54% increase in probability across the lower half of its range (0.08–0.38), but a 211.84% decrease in the upper half (0.38–0.53). This pattern implies that intermediate PSL values support murrelet presence. Tidal currents exhibited a modest 35.75% increase in probability across the lower half of its range (-0.01–0.71), followed by a significant 254.96% decrease in the upper half (0.71–0.99). This suggests that low to moderate tidal currents are preferable during the nonbreeding season, with stronger currents likely reducing the probability of occurrence. SHOREdist was associated with an 81.59% decrease in probability across the lower half of its range (1,395.24–8,028.12 m), and a 51.53% decrease in the upper half (8,028.12–15,293.26 m). This reinforces the importance of proximity to shorelines for murrelets during the nonbreeding season, with the probability of occurrence declining as the distance from shore increases. Survey type had a significant effect on probability of occurrence during the nonbreeding season, with lower occurrence related to the shoreline surveys. NESTcohweigh, NESTcoh, STREAMdist, slope and NPGO had an insignificant effect on probability of murrelet occurrence during the nonbreeding season (S3 Table).

The predictive maps (Figs 3 and 4) indicating the probability of presence for murrelets during the breeding and nonbreeding season had many similarities, but some important distinctions. For the breeding season map, we found that the highest probability of presence (0.85–1) was predicted to be around Desolation Sound area, Jervis Inlets, around Northwest Texada, Comox Sound, and around the waters of Sidney and Victoria. In contrast, during the nonbreeding season, we found that high probability of presence also arose mainly around Comox Sound and Desolation Sound, however, the predicted occurrences appearing more dispersed overall.

We compared the predictive performance of shoreline and marine surveys during both the breeding and nonbreeding seasons (S1 File). During the breeding season, models trained on marine data provided moderate predictive accuracy when applied to shoreline data, but the reverse showed significantly lower accuracy, highlighting the limitations of shoreline surveys in capturing marine variability. In contrast, during the nonbreeding season, the predictive capability between shoreline and marine surveys was more balanced, likely due to better data coverage or more even distribution of murrelets during the nonbreeding season. These findings underscore the importance of considering the type of survey data used in habitat modeling. For a detailed analysis, including cross-validation results and individual predictive maps and partial plot comparisons, please refer to the S1 File in the Supporting Information section.

## Discussion

In this study, we examined factors influencing murrelet distribution during both breeding and nonbreeding seasons within the Salish Sea region of Canada. We expected that murrelets would select marine areas that optimize energy expenditure during the breeding season by staying close to abundant and intact potential nesting habitats, and that this relationship would be less pronounced during the nonbreeding season. Our findings provided mixed support for our expectations, and revealed some unexpected patterns in the relationship between murrelet presence and nesting habitat. During the breeding season, while the association with larger and more cohesive potential nesting areas was significant, the relationship was somewhat unclear due to the varied patterns observed. Specifically, the relationship with NESTcohweight showed an initial decrease in occurrence probability followed by an increase, suggesting a complex and non-linear relationship that requires further exploration. Similarly, the association with NESTarea during the breeding season was not linear, with an initial decrease in occurrence probability across smaller nesting areas followed by an increase for larger areas. These patterns indicate that while nesting habitat is important, the exact nature of its influence on murrelet distribution during the breeding season is not straightforward and may involve additional factors or thresholds not fully captured in our models.

Interestingly, during the nonbreeding season, the relationship between murrelet presence and nesting habitat area had higher predictive performance and was more consistent. This was somewhat surprising, as we had anticipated a less pronounced relationship due to the murrelets’ reduced reliance on nesting sites outside of the breeding period. However, the data suggest that larger nesting areas continue to play a significant role in murrelet distribution during the nonbreeding season, perhaps due to the continued availability of resources or the benefits of familiar territory. Additionally, our results underscored the importance of prey availability, particularly Pacific sand lance (PSL) habitat quality, in influencing murrelet distribution. During both the breeding and nonbreeding seasons, murrelet presence was associated with intermediate PSL habitat quality, indicating a preference for foraging in areas where high-quality prey patches are surrounded by lower-quality areas, possibly to optimize foraging efficiency. The variability in habitat use patterns during the nonbreeding season, likely driven by a broader diet [65] and reduced nesting site dependency, made it more challenging to predict murrelet presence solely based on environmental covariates, which was expected. To further understand these unexpected patterns, we will explore the underlying factors influencing murrelet distribution in the discussion below.

Between 1998 and 2001, murrelets were tagged and tracked using radio telemetry in the remote locations of Desolation sound (in the Salish Sea) and Clayoquot Sound (on the west coast of Vancouver Island). A recent comparative analysis was completed which found that birds in Clayoquot Sound nested closer to foraging sites than in Desolation sound [11]. The area of Clayoquot Sound is characterized by relatively intact old growth forests, whereas Desolation Sound has experienced more forest harvest [35]. These past studies support the relationship between the distance to intact potential nesting habitat to where murrelets forage. Considering nesting, it’s logical that murrelets would prefer to be as close as possible to their foraging locations, particularly for chick-provisioning adults. Potential nesting habitat around the same areas in the Salish Sea is becoming increasingly fragmented [35,66]. Fragmentation increases the risk in predation, and decreases nest site use [28]. During the breeding season, our observations revealed an influence of distance-weighted nesting habitat cohesion on murrelets. When the index values of this covariate were low, indicating habitats situated far from their breeding grounds and characterized by fragmentation, our data pool was limited, and the confidence intervals for the corresponding range were notably wide for corresponding predictions. Conversely, in areas with a higher number of observations available for model building, a slight positive relationship emerged between the probability of murrelet occurrence and proximity to unfragmented nesting habitats. However, from these data it is clear the relationship to nesting habitat is complex. Garcia-Heras (2024) found that during poor ocean conditions, murrelets decoupled their marine habitat use from proximity to old-growth nesting habitat, selecting areas near small estuaries and localized upwelling instead, while exhibiting limited overlap with protected marine areas [67]. Though our work could not explore these types of interactions fully, future work should expand survey coverage over time to allow for the exploration on how climate and nesting habitat interactions influence habitat selection in Canadian waters. For the nonbreeding season, we observed an intriguing relationship concerning the amount of habitat within an 80 km radius, with a stronger than expected positive relationship to potential nesting areas during the nonbreeding season. Generally, a positive association was not entirely unexpected, aligning with observations of birds visiting nesting sites during the nonbreeding season [68]. However, our study marks the first instance of such an association being measured in murrelet nonbreeding populations, indicating the year-round role potential nesting sites play in murrelet habitat associations.

Pacific sand lance are an important food source for murrelets in the Pacific Northwest [18,69]. Previous efforts that have related murrelets to Pacific sand lance have positively related coarse grain sand, which is associated with Pacific sand lance, to murrelet presence during the breeding season [12,13]. Recently developed Pacific sand lance quality habitat layers gave us a chance for the first time to investigate how tightly murrelets on the water relate to the habitat of their favored prey species. In our results, we observed a correlation between the presence of murrelets and intermediate likelihood of Pacific sand lance habitat for both breeding and nonbreeding seasons. The original Pacific sand lance layer was meticulously calculated at a fine scale (20–50 m grid size), yet in our analysis, we derived the average value over a 3 by 3 km radius. This broader scope might account for the selection of more intermediate values, possibly due to pooling and averaging PSL habitat predictions across a larger spatial scale. Interestingly, the relationship is somewhat more pronounced in the winter than in the summer. Initially, this year-round association was surprising, since in the winter, when water temperatures are colder and food availability is lower, Pacific sand lance may spend more time burrowed in the substrate to conserve energy [19,70]. However, this association is consistent with findings that older sand lance return to the water column by the end of March [71], which coincides with our definition of the murrelet nonbreeding season from September to the end of March. The continuity of this association to Pacific sand lance habitat throughout the year is supported by the observation of similar hotspot locations for murrelets during summer and winter overlapping with sand lance subtidal habitat near Sidney, British Columbia [22,72].

Another potential reason for observing more intermediate associations with Pacific sand lance habitat is the broad diet potential of murrelets. Areas that not only have pockets that support quality sand lance habitat, but also other potential prey may be a strategic preference. We recognize that, although vital to murrelets in this region, Pacific sand lance are not the only potential prey item that murrelets might target in this area [73–75]. Murrelets are known to feed on a wide variety of prey, depending on time of year and prey species availability, including northern anchovies (*Engraulis mordax*, [75]), shiner perch (*Cymatogaster aggregata*), Pacific sandfish (*Trichodon trichodon*), and smelt (*Osmeridae*) [76], as well as *Euphausiid crustacea*. Additionally, within the Salish Sea, juvenile herring and Pacific sand lance often school together in the Haro Strait region in known hot spots for murrelets and other alcids [72]. For these reasons, there is a need for comprehensive spatial and temporal distribution maps for additional prey items, such as juvenile herring, juvenile salmonids, and northern anchovy.

In marine ecosystems, proximity to shorelines with freshwater inputs can foster conditions conducive to nutrient-rich waters, promoting productivity by supplying essential nutrients to surface waters that sustain the growth of phytoplankton and other primary producers [77]. A key driver of this dynamic is the convergence of freshwater and saltwater in specific areas, resulting in heightened productivity and offering more foraging opportunities compared to locations lacking such convergence [20]. Furthermore, areas situated closer to the shore and in proximity to streams offer the added advantage of proximity to flyway zones, frequently utilized by murrelets for accessing nesting sites [11]. This correlation has been established in various breeding season murrelet studies [12,24]. We found that the breeding season portion of our analyses supports these previous findings. Consequently, our results underscore these as pivotal features associated with murrelets during their breeding period, reinforcing the importance of these ecological elements. The association with proximity to shore and streams influenced our habitat quality maps, emphasizing locations within inlets. Inlets may play a crucial role as flyway zones for murrelets transporting food to their nests. Supporting evidence is derived from radar monitoring stations that count murrelets flying through Bute and Toba Inlet [26]. Additionally, our study revealed that inlets where murrelets were not predicted to occur had less potential nesting habitat around them. This provides additional evidence supporting the notion that inlet use may be connected to nesting access. An intriguing anomaly is evident in the vicinity of Howe Sound and Burrard Inlet, where murrelet activity persists throughout the year, with notably higher numbers during the nonbreeding season (see S5 Fig). Despite this, our predictive models indicate a low likelihood of murrelet presence in these regions (Figs 3 and 4). The scarcity of nesting sites in the Vancouver area significantly contributes to this overall classification of low occurrence probability. Nonetheless, pockets of undisturbed, potential nesting habitats remain in the vicinity, potentially supporting a few nesting pairs during the summer months. Furthermore, there may exist additional, unaccounted sources of winter prey for murrelets in the area.

Our study took place within two marbled murrelet conservation zones: the Southern Mainland Coast and East Vancouver Island. While our analysis considered the overall influence of environmental covariates on murrelet probability of occurrence, it is important to note that murrelets may be selecting their marine habitat areas based on different attractions, as each zone presents unique advantages and challenges that influence their nesting and foraging behaviors (S4 Table). The Southern Mainland Coast region, characterized by its extensive land, numerous deep inlets, and steep bathymetric slopes, offers a diverse and dynamic foraging environment. These deep marine areas are likely productive due to their complex topography, although the region lacks the cooler, nutrient-rich inflows from the Pacific Ocean that benefit East Vancouver Island. Moreover, the Southern Mainland Coast has less tidal mixing, which may result in lower overall nutrient availability compared to East Vancouver Island. Despite these disadvantages, the Southern Mainland Coast’s larger overall habitat area and its varied marine environments could attract murrelets seeking a wide range of nesting and foraging opportunities, even in the face of higher human population density and associated disturbances [39]. In contrast, East Vancouver Island benefits from cooler, nutrient-rich waters and stronger tidal mixing [40], and support higher quality Pacific sand lance habitat (S4 Table). When comparing the survey type interactions (S1 File) we observed with the shoreline surveys, a higher probability of occurrence was predicted for stronger tidal current values. This supports the notion that close to shore tidal currents are providing nutrient mixing that is beneficial to murrelets. The area’s gentler bathymetric slopes and shallower waters potentially provide more predictable and stable foraging conditions. However, there is less overall available potential nesting area’s on East Vancouver Island, which may limit the number of available nesting sites and increase competition among murrelets. These contrasting features suggest that different aspects of each region may attract murrelets, with the Southern Mainland Coast having more extensive and varied habitats, while East Vancouver Island potentially provides higher quality foraging grounds.

Our study should be interpreted with the knowledge that we utilized multiple datasets by different sources and survey types, and it was not possible to explicitly model detectability. The inherent variability in the data collection methods across different surveys and the lack of a unified approach to detecting murrelets may introduce biases that could affect the results. However, we addressed these challenges by applying suitable statistical techniques and supplementing our analysis with additional results to investigate these potential biases. Various regions exhibit significant annual and interannual variability in density and abundance [78,79], which was why we used year as a random variable, adding confidence to our results. Murrelets have been found to persistently use the same marine areas over time, even in locations where year-to-year abundance variability has been documented [12,78]. Additionally, we could not incorporate potential nesting habitat information from adjacent Washington State, USA, into our analyses, which could influence the outcomes. Habitat quality models developed for Washington State, predicting potential nesting habitat for murrelets, differ from those used in Canada [80]. Consequently, our analysis relied solely on data from Canada to model murrelet distribution and did not consider the potential impact of potential nesting habitat in adjacent areas in Washington. Our focus was on static covariates to create a baseline understanding between the breeding and nonbreeding season. However, we emphasize the importance other variables can play, such as dynamic oceanographic components [8] and social behavioral [81] on predicting distribution and abundance of this secretive species. Our modelling work provides a repeatable methodological baseline for future studies to identify and conserve murrelet marine habitats.

### Management implications

These findings are distinctive in illustrating distribution variations within a region for this threatened species during breeding and nonbreeding seasons. Conservation planning should account for both the overall quantity of terrestrial habitat and the quality of proximate marine habitat [82]. Predictive maps further highlight differences in inlet use between summer and winter, underscoring the significance of these areas during the breeding period. A comparison with the presently designated critical habitat for murrelets in the Salish Sea reveals substantial overlap, yet notable differences exist, especially in the southern Salish Sea region around Sidney and Victora [7]. This work establishes a benchmark for future research, offering opportunities to enhance our understanding of the species’ distribution and habitat use and needs, throughout the year. Our findings are relevant for ongoing initiatives aimed at characterizing and safeguarding critical marine habitats for this threatened, high-profile seabird.

## Supporting information

S1 Fig Potential marbled murrelet nesting habitat and quality Pacific sand lance habitat. (JPG)

S2 Fig Breeding season correlogram plots. Correlograms showing Moran’s I values over a range of distance lags (at 1000-m intervals) for raw counts (RAW), residuals of model using only predictor covariates (ENVIRONMENTAL FACTORS) and residuals of model using coordinates and covariates (LAT/LON).
(PNG)

S3 Fig Marginal effects of key environmental covariates on the probability of Marbled Murrelet occurrence during the breeding season displaying transformed and untransformed values. Each plot shows the predicted probability of occurrence as a function of the transformed values of each covariate (bottom x-axis), with the corresponding untransformed values shown on the top x-axis. Covariates include NESTarea (Potential Nesting Habitat Area), NESTcohweight (Nesting Habitat Cohesion Index), PSL (Pacific Sand Lance Habitat), Tidal currents, STREAMdist (Distance to Streams), and SHOREdist (Distance to Shoreline).
(PNG)

S4 Fig Marginal effects of significant environmental covariates on the probability of Marbled Murrelet occurrence during the nonbreeding season displaying transformed and untransformed values. Each plot shows the predicted probability of occurrence as a function of the transformed values of each covariate (bottom x-axis), with the corresponding untransformed values shown on the top x-axis. Covariates include NESTarea (Potential Nesting Habitat Area), PSL (Pacific Sand Lance Habitat), Tidal currents, and SHOREdist (Distance to Shoreline).
(PNG)

S5 Fig Marine habitat quality maps and observed occurrences. Probability of murrelet presence overlayed with presence points (Centroid), for both the breeding (left) and nonbreeding (right) seasons.
(JPG)

S1 Table Survey and contributor information for datasets used in study. (XLSX)

S2 Table Concurvity paired values for the breeding and nonbreeding datasets. (CSV)

S3 Table GAM Results for the marbled murrelet probability of presence during the breeding and nonbreeding season. Numbers are the EDFs (estimated degrees of freedom); higher numbers indicate relationship is more complex and lower numbers means relationship is approaching linearity. Bolded terms has a P-value less than 0.05 and F represents factor covariates. If no values are displayed, they were selected out of the model.
(CSV)

S4 Table Summary of environmental variables by marbled murrelet conservation zones: East Vancouver Island and Southern Mainland Coast. (PNG)

S1 File (PDF)

S2 File (PDF)
