# Supplementary figures and images for: Predicting marine habitat for marbled murrelets during breeding and nonbreeding seasons in the Salish Sea, British Columbia, Canada
Source: PLoS One. 2025 Jan 16;20(1):e0316946. doi: 10.1371/journal.pone.0316946 (PMC11737741; doi:10.1371/journal.pone.0316946)

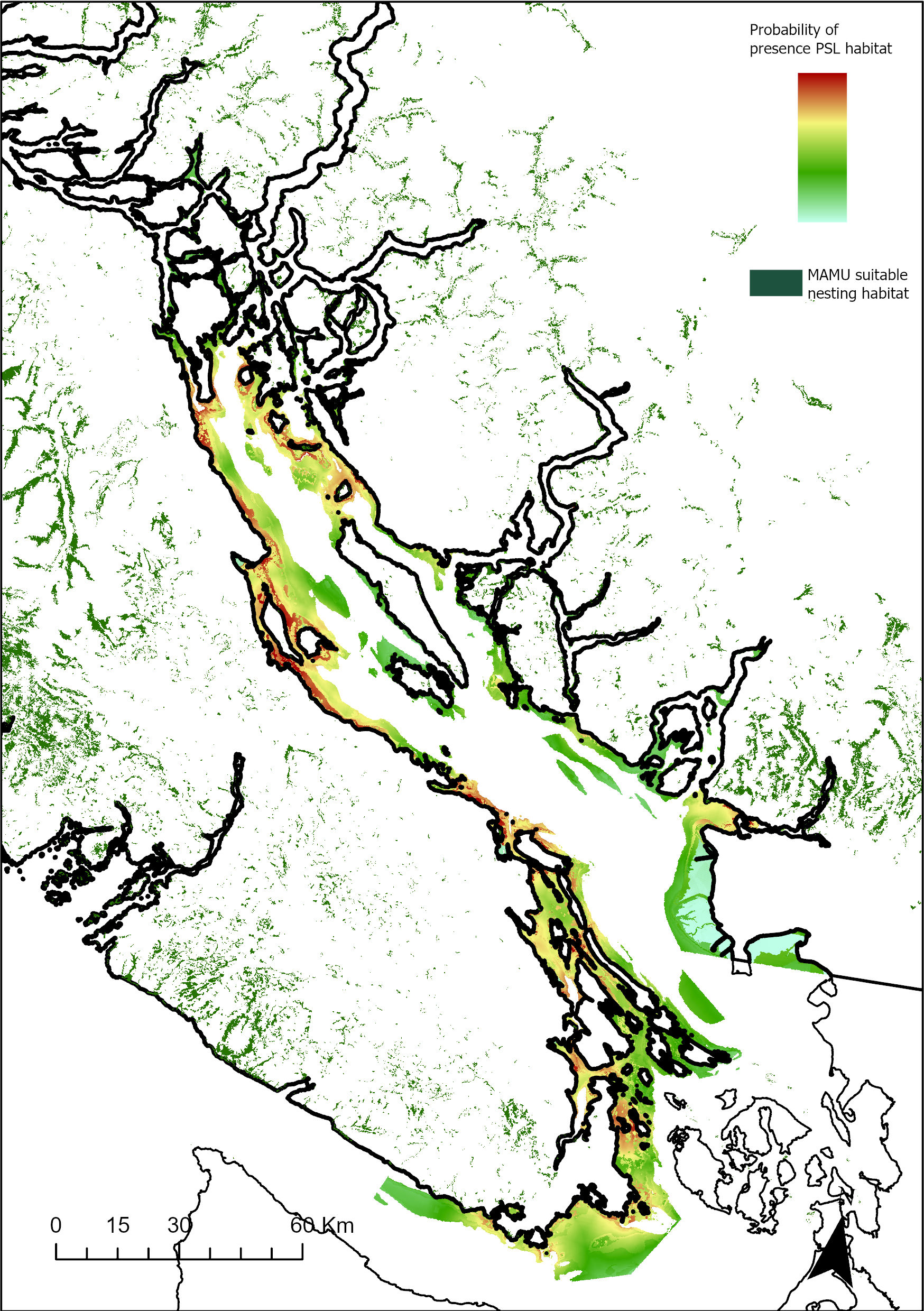

Supplement: S1 Fig — (JPG) [file pone.0316946.s001.jpg]

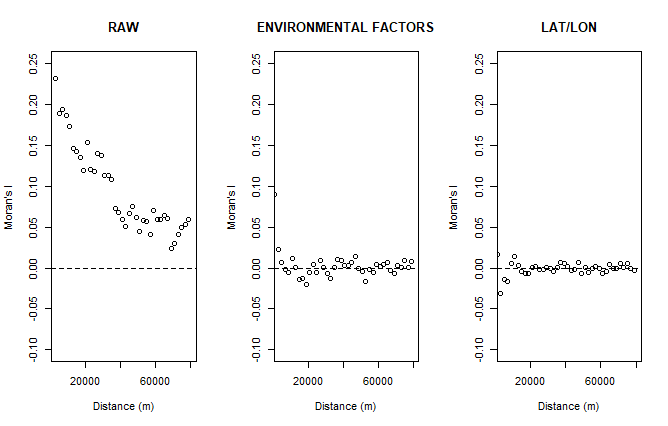

Supplement: S2 Fig — Correlograms showing Moran’s I values over a range of distance lags (at 1000-m intervals) for raw counts (RAW), residuals of model using only predictor covariates (ENVIRONMENTAL FACTORS) and residuals of model using coordinates and covariates (LAT/LON). (PNG) [file pone.0316946.s002.png]

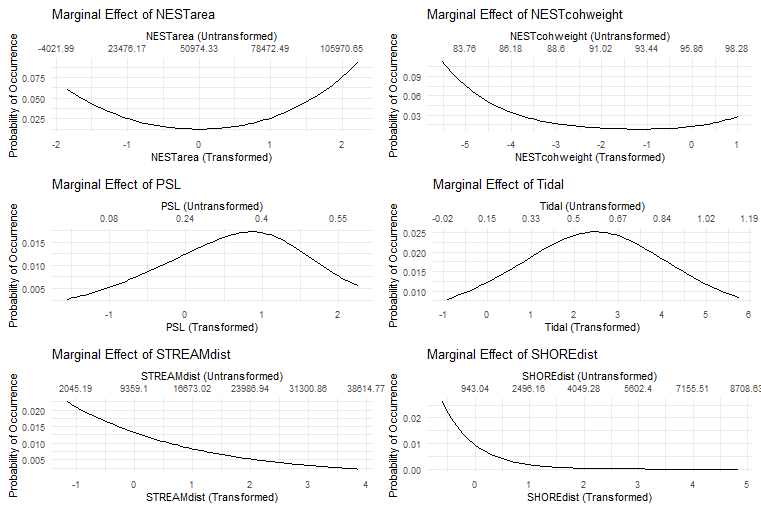

Supplement: S3 Fig — Each plot shows the predicted probability of occurrence as a function of the transformed values of each covariate (bottom x-axis), with the corresponding untransformed values shown on the top x-axis. Covariates include NESTarea (Potential Nesting Habitat Area), NESTcohweight (Nesting Habitat Cohesion Index), PSL (Pacific Sand Lance Habitat), Tidal currents, STREAMdist (Distance to Streams), and SHOREdist (Distance to Shoreline). (PNG) [file pone.0316946.s003.png]

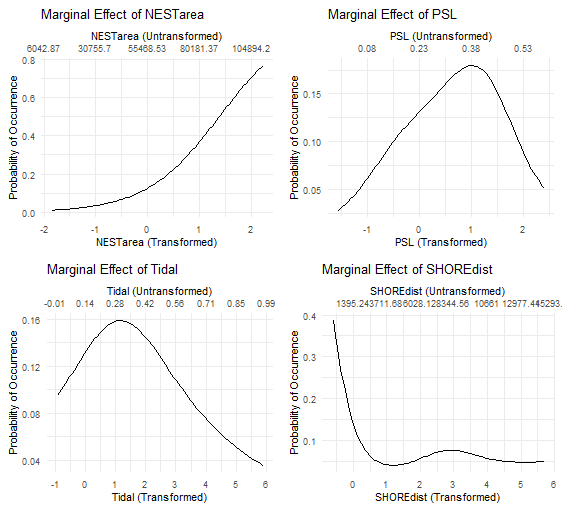

Supplement: S4 Fig — Each plot shows the predicted probability of occurrence as a function of the transformed values of each covariate (bottom x-axis), with the corresponding untransformed values shown on the top x-axis. Covariates include NESTarea (Potential Nesting Habitat Area), PSL (Pacific Sand Lance Habitat), Tidal currents, and SHOREdist (Distance to Shoreline). (PNG) [file pone.0316946.s004.png]

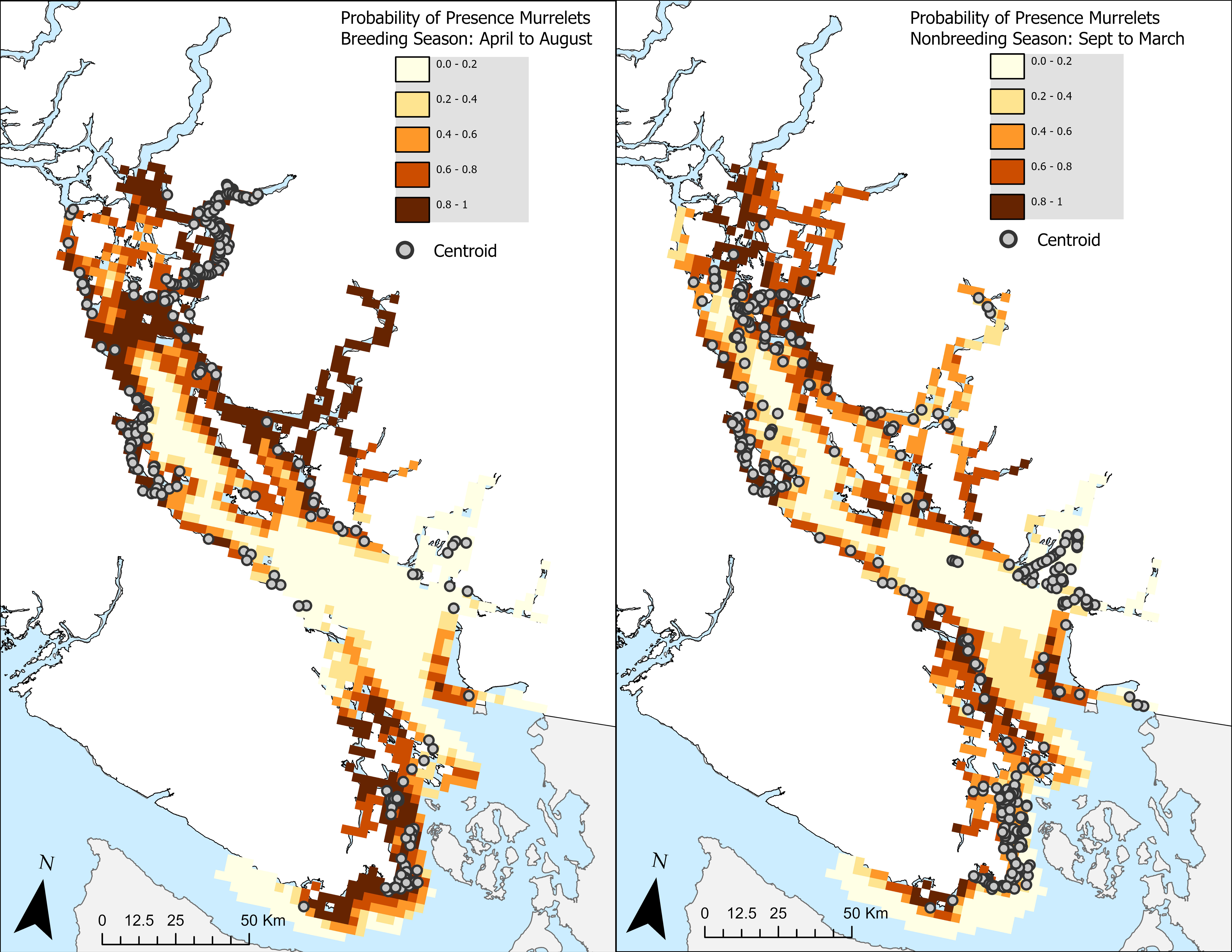

Supplement: S5 Fig — Probability of murrelet presence overlayed with presence points (Centroid), for both the breeding (left) and nonbreeding (right) seasons. (JPG) [file pone.0316946.s005.jpg]

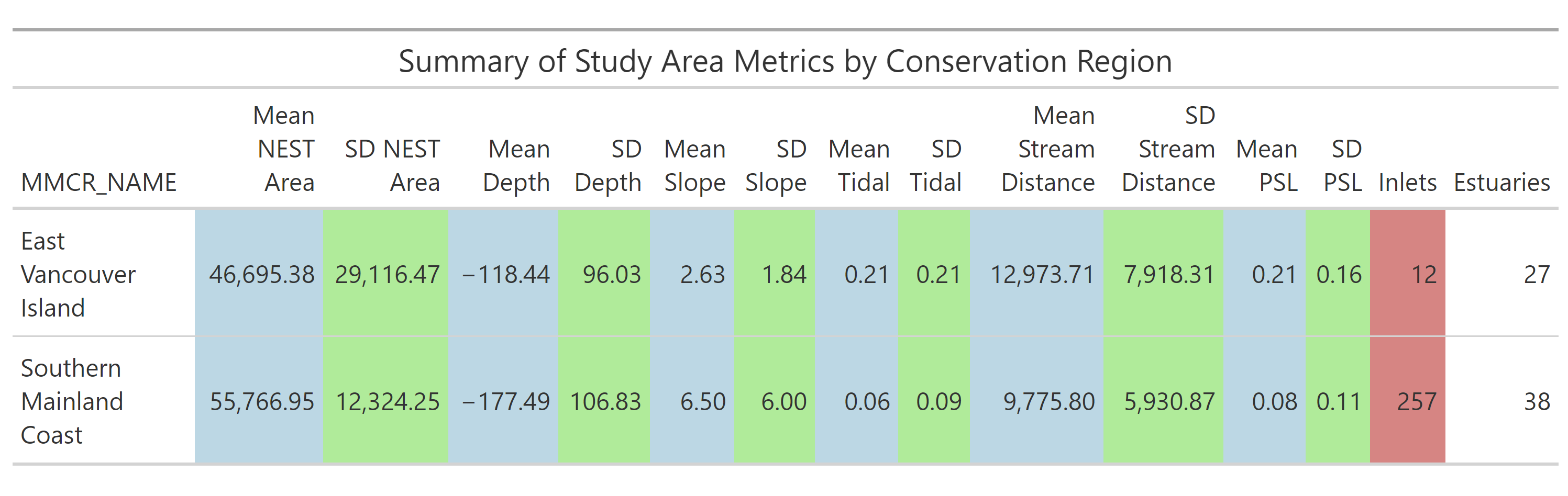

Supplement: S4 Table — (PNG) [file pone.0316946.s009.png]
