# Supplementary material for: Predicting marine habitat for marbled murrelets during breeding and nonbreeding seasons in the Salish Sea, British Columbia, Canada
Source: PLoS One. 2025 Jan 16;20(1):e0316946. doi: 10.1371/journal.pone.0316946 (PMC11737741; doi:10.1371/journal.pone.0316946)
Supplement: S1 File — (PDF) [file pone.0316946.s010.pdf]

S1 File: Comparison of Shoreline and Marine Surveys

Seabird surveys that include presence and absence data are limited within the Canadian portion of the Pacific Northwest, particularly during the breeding season of marbled murrelets. We aimed to utilize reliable, well-established surveys to inform the habitat suitability of this Species at Risk. However, the coverage of shoreline surveys does not align with that of marine surveys, leading to gaps in data when considered individually. While combining shoreline and marine surveys provides a more comprehensive understanding, shoreline surveys alone are not effective predictors of habitat suitability for the Salish Sea.

To address the discrepancies in survey structures, we included "Survey Type" as a covariate in our analyses. Despite this adjustment, caution is still necessary. It is challenging to make direct comparisons between surveys with such different ranges and overall frequency. Additionally, shoreline surveys are confounded by variables like distance to shore and proximity to potential nesting habitats, which are distance-weighted. Although we cannot fully resolve these issues, we have aimed for transparency by providing this additional model with survey type set as an interactive term for the relevant covariates. This section contains prediction and partial prediction plot comparisons and cross-validation analyses between the two survey types.

We encourage readers to explore these additional plots, which allow for a more nuanced comparison and understanding of the effects these different survey types have on the overall analysis.

Summary of Results:

Breeding Season:

The cross-validation results underscore the challenges of using shoreline data to predict marine survey outcomes and vice versa. The ROC and PRC values indicate that models trained solely on marine data provide moderate predictive accuracy when applied to shoreline data. However, the reverse—training on shoreline data and predicting marine outcomes—shows significantly lower predictive accuracy, as reflected in the lower ROC and PRC scores. This suggests that while combining data from both survey types can enhance overall understanding, caution should be exercised when interpreting results from models based exclusively on one type of survey data. Shoreline surveys, in particular, may not adequately capture the variability inherent in marine environments during the breeding season, which could be attributed to the limited coverage and amount of shoreline data available during this time. The lack of coverage likely influences the poor predictive power of shoreline surveys on their own.

The partial plot comparisons between the shoreline and marine GAM outputs reveal both differences and similarities. The differences primarily stem from the inherent spatial differences associated with each survey type. The most pronounced differences were observed for covariates like nesting cohesion distance-weighted (NESTcohweight) and distance to streams (STREAMdist), which are influenced by proximity to shorelines. NESTcohweight is significant only in marine surveys during the summer season, indicating that its influence on Marbled Murrelet presence is more pronounced in marine environments, likely due to the inherent spatial differences and limited shoreline data, which reduce the ability to predict marine outcomes using shoreline data. Similarly, STREAMdist is significant only in marine surveys during the summer season, playing a crucial role in determining Marbled Murrelet presence in these environments, a relationship less evident in shoreline data. Conversely, similarities were observed in variables less influenced by distance to land, such as tidal currents and NPGO. However, tidal conditions are significant only in shoreline surveys during the summer season, where proximity to land intensifies their effects. PSL (Pacific sand lance) also reflects its critical role in areas closer to shore during the breeding season, as it is significant only in shoreline surveys, though its significance in marine surveys is less pronounced, possibly due to the spatial differences in survey types. The relationship shape in the partial plots were similar between both survey types for PSL.

There is over 50% less data available for the shoreline surveys, with even fewer of those points located within the northern region of the Salish Sea. Overall coverage should also be considered when interpreting these results.

Nonbreeding Season:

During the nonbreeding season, the predictive capability between shoreline and marine surveys is notably balanced, as confirmed by cross-validation results. Both survey types yield similar outcomes, with ROC and PRC scores reflecting comparable predictive accuracy. This balanced performance is further supported by the partial plots, which generally illustrate consistent relationships between key covariates across both survey types, despite some differences.

For instance, NESTarea shows a more complex relationship with marbled murrelet presence, particularly in shoreline surveys where the influence of nesting area size is less pronounced compared to marine surveys. Despite this, the overall trend is similar, with both survey types indicating a weaker association with smaller nesting areas. NESTcohweight displays a generally consistent shape across both survey types; however, in shoreline surveys, there is a sharper drop-off, indicating that higher nesting cohesion is associated with a lower probability of Marbled Murrelet occurrence in these environments during the nonbreeding season. This

suggests that while NESTcohweight plays a role in both environments, its influence is more pronounced in shoreline surveys. PSL (Pacific sand lance) maintains a consistent shape across both survey types, with a peak relationship at mid-range values, though marine surveys show a slightly broader and more pronounced peak. This similarity suggests that PSL is a critical factor across both environments during the nonbreeding season. On the other hand, STREAMdist had an insignificant relationship with marbled murrelet presence in both survey types, indicating that distance to streams is not a key factor during the nonbreeding season. Tidal conditions are influential in both shoreline and marine surveys, though with slightly differing shapes. Shoreline surveys reveal a peak at mid-range tidal values, whereas marine surveys depict a broader influence across a range of values, suggesting that tidal conditions are consistently important in both environments, albeit with slight variations in their impact. Slope and NPGO demonstrate minor differences between the survey types, with Slope having a somewhat stronger influence in marine surveys, and NPGO showing a modestly more positive relationship in shoreline surveys. These differences, though present, are not as pronounced, indicating that the overall environmental gradients affecting Marbled Murrelet presence are relatively consistent across different survey types.

Overall, the nonbreeding season analysis reveals a more balanced and consistent predictive relationship between shoreline and marine surveys. While some covariates exhibit variations in their influence, the general patterns observed across the partial plots suggest that both survey types capture similar environmental signals, leading to comparable predictive accuracy across the two environments.

Conclusion:

Combining both shoreline and marine surveys offers a more holistic assessment of marbled murrelet habitat quality, covering both nearshore and offshore environments. Given that these environments are interconnected, analyzing them together ensures that potential habitat overlaps and interactions are not missed. However, the results clearly indicate that reliance on a single survey type, whether shoreline or marine, could lead to incomplete or misleading interpretations of habitat suitability.

Ideally, having a consistent survey type across both space and time would provide the most reliable and comprehensive data. However, we are working with available data from various sources, which introduces certain limitations.

Model with Interactive Terms for Survey Type:

Breeding dataset

$$\text{logit}(y_i) = \beta_0+s(\text{Longitude}_i,\text{Latitude}_i,k=100) +s(\text{NESTarea}_i,\text{by}=\text{SurveyType}_i) +s(\text{NESTcohweight}_i)+s(\text{PSL}_i,\text{by}=\text{SurveyType}_i)+s(\text{STREAMdist}_i ,\text{by}=\text{SurveyType}_i)+s(\text{SHOREdist}_i)+s(\text{Tidal}_i,\text{by}=\text{SurveyType}_i)+s(\text{NPGO}_i)+\text{SurveyType}_i+\text{Inlets}_i+u(\text{SurveyID}_i)+v(\text{Year}_i)+\text{offset}(\log(\text{Effort}_i))$$

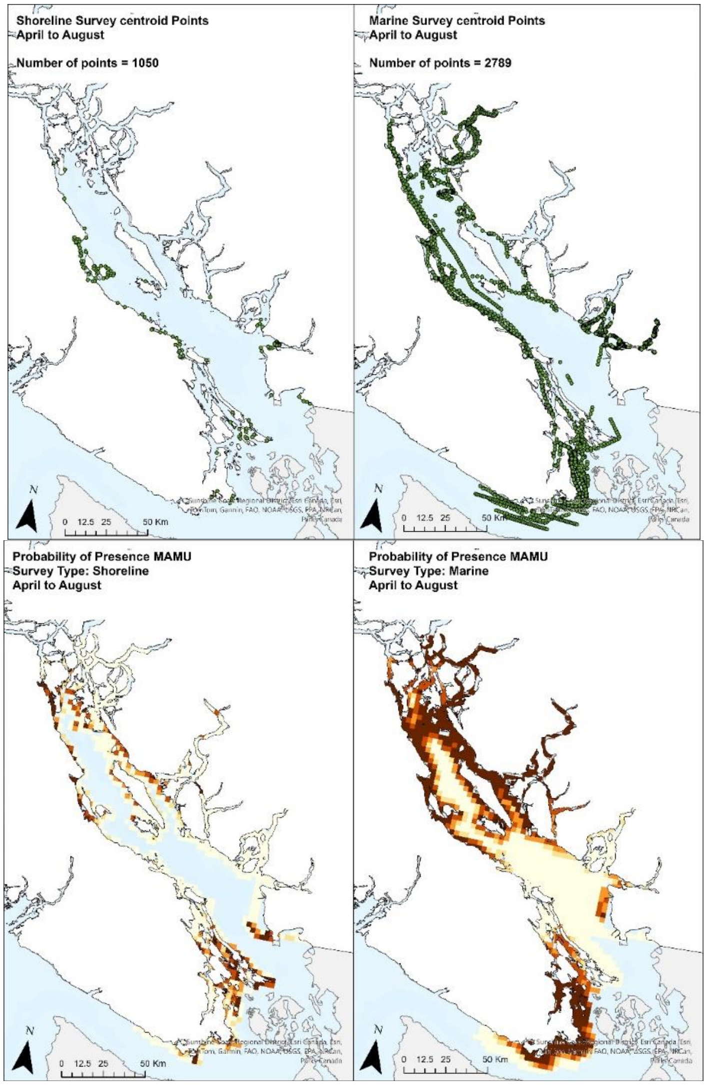

Fig A: **Raw points and subsequent predictive maps using interactive model with Survey Type Set to Shoreline Surveys (left) or Marine surveys (Right) during the breeding season (April to August)**: Predictions are restricted to within 3 km of the shoreline for the shoreline survey prediction map.

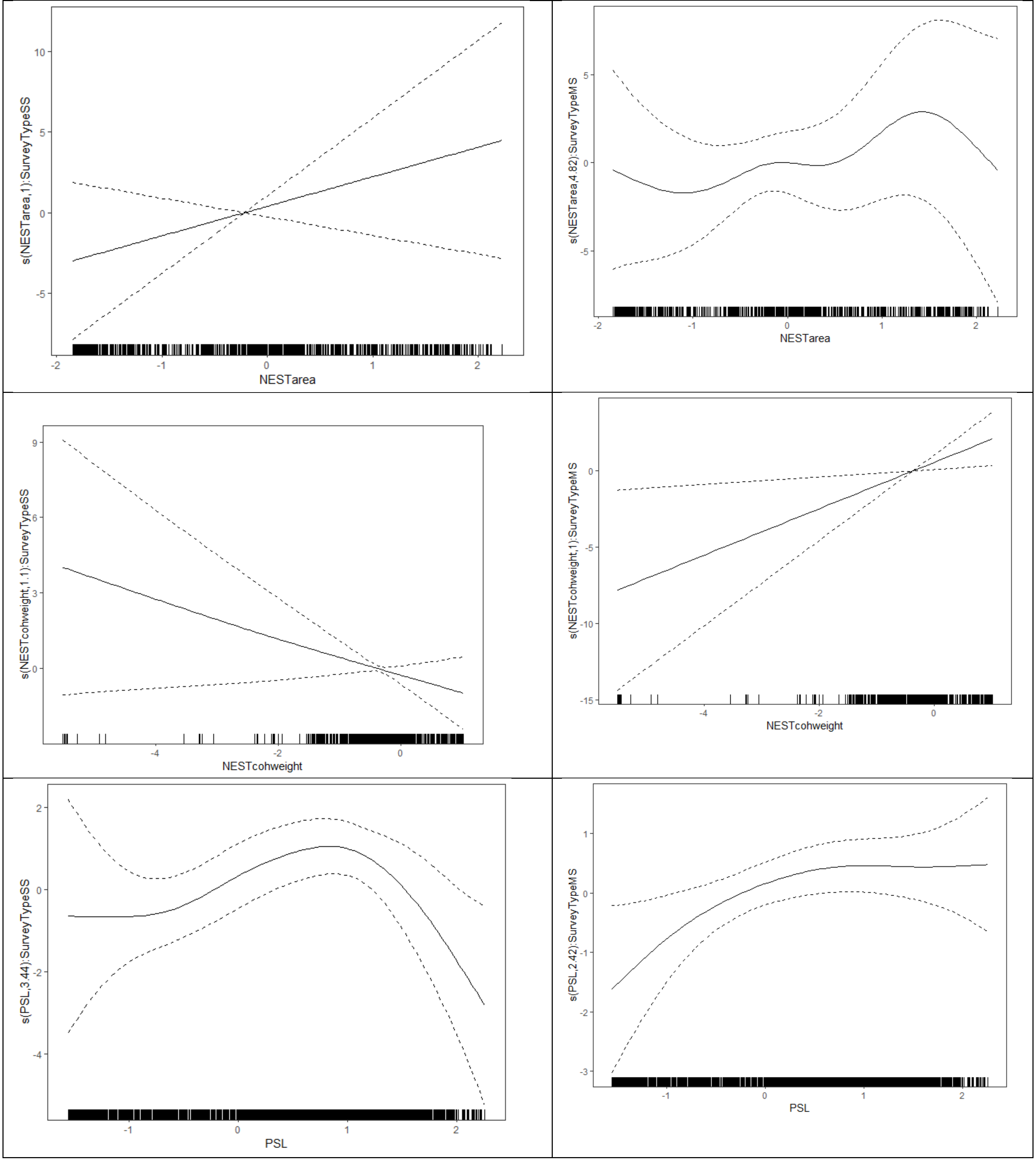

s(NESTarea,1):SurveyTypeSS
10
5
0
-5
-2
-1
0
1
2
NESTarea
s(NESTarea,4.82):SurveyTypeMS
5
0
-5
-2
-1
0
1
2
NESTarea
s(NESTcohweight,1.1):SurveyTypeSS
9
6
3
0
-4
-2
0
NESTcohweight
s(NESTcohweight,1):SurveyTypeMS
0
-5
-10
-15
-4
-2
0
NESTcohweight
s(PSL,3.44):SurveyTypeSS
2
0
-2
-4
-1
0
1
2
PSL
s(PSL,2.42):SurveyTypeMS
1
0
-1
-2
-3
-1
0
1
2
PSL

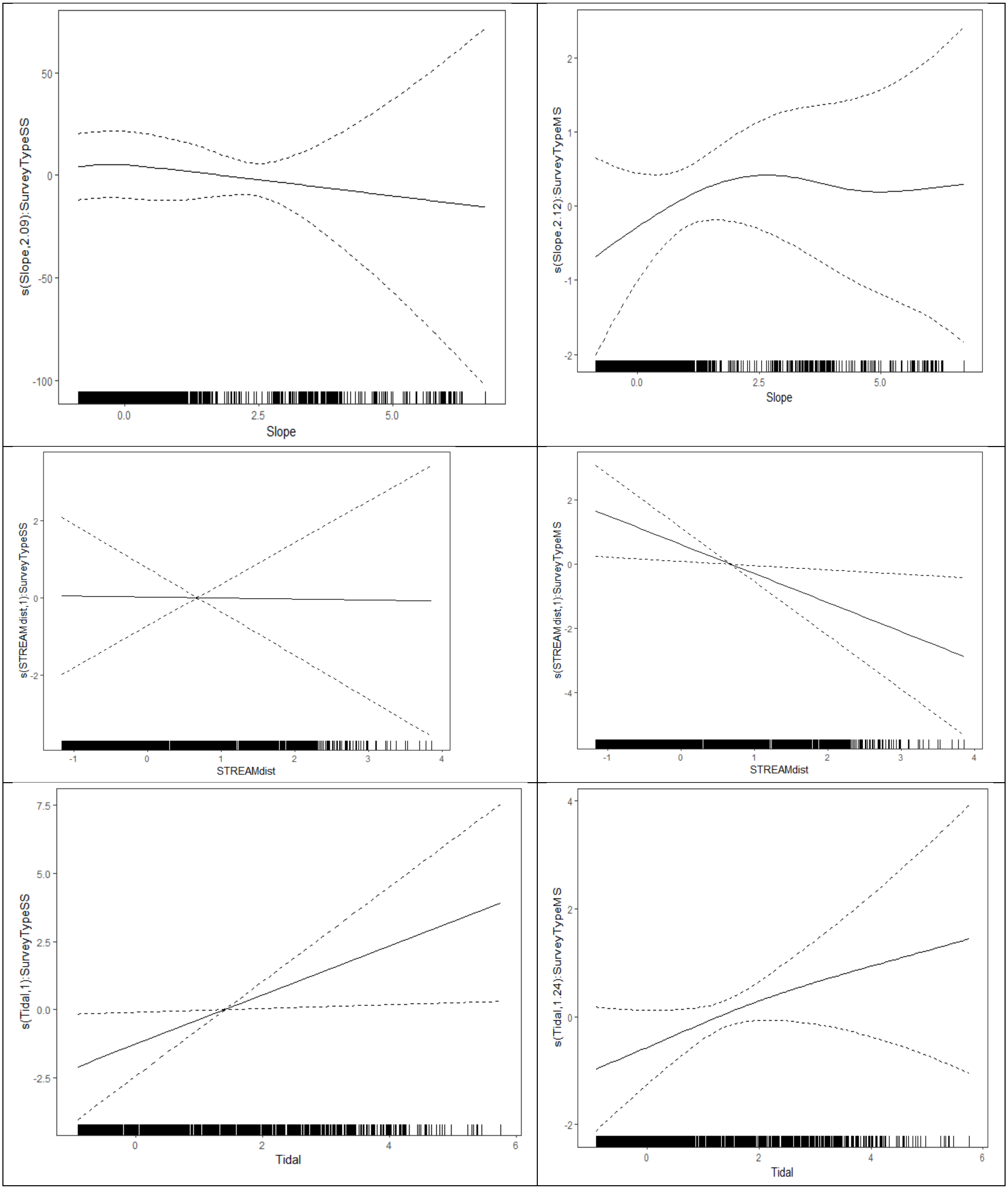
s(Slope,2.09):SurveyTypeSS
Slope
s(Slope,2.12):SurveyTypeMS
Slope
s(STREAMdist,1):SurveyTypeSS
STREAMdist
s(STREAMdist,1):SurveyTypeMS
STREAMdist
s(Tidal,1):SurveyTypeSS
Tidal
s(Tidal,1.24):SurveyTypeMS
Tidal

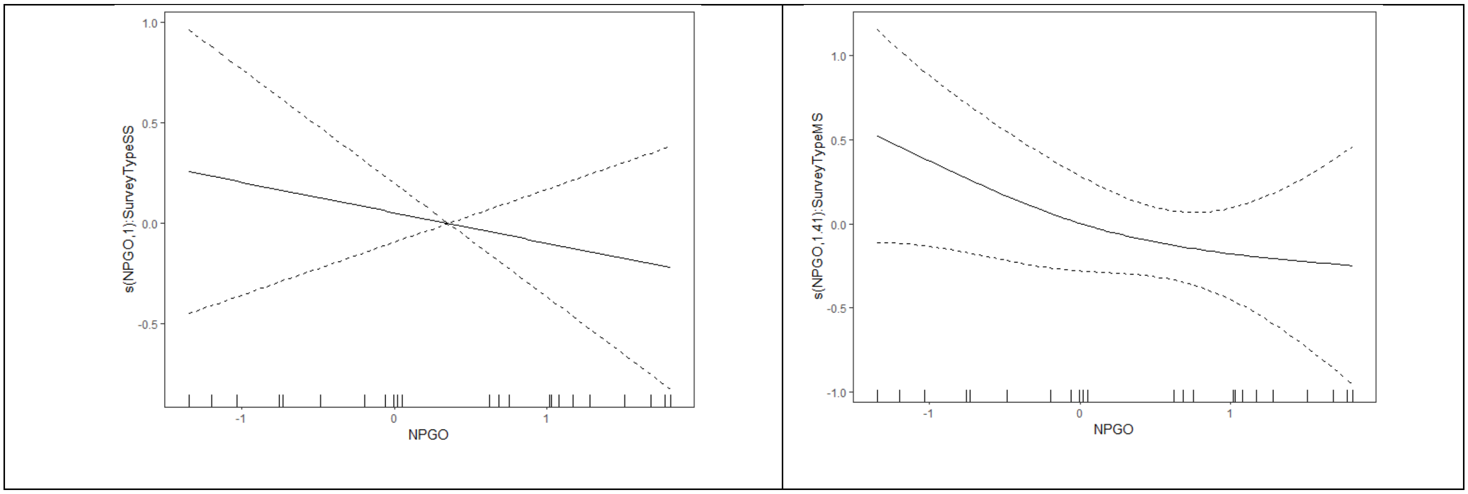

Fig B: Partial plot comparisons of marine and shoreline GAM outputs for the breeding season dataset.

Table A: Summary of significant covariates for marbled murrelet presence during the breeding season, based on Generalized Additive Model (GAM) outputs. The table presents the significance levels and p-values for each covariate, comparing their effects in marine surveys (MS) and shoreline surveys (SS).

Covariate	Marine (MS)	Shoreline (SS)	Explanation
NESTcohweight	Significant (p=0.02)	Not Significant	NESTcohweight is significant only in marine surveys during the summer season when considered separately.
PSL	Not Significant	Significant (p=0.01)	PSL is significant only in shoreline surveys during the summer season when considered separately.
STREAMdist	Significant (p=0.02)	Not Significant	STREAMdist is significant only in marine surveys during the summer season when considered separately.
SHOREdist	Significant (p=<2e-16)	Significant (p=<2e-16)	SHOREdist shows a consistent significant effect across both survey types.
Tidal	Not Significant	Significant (p=0.03)	Tidal conditions are significant only in shoreline

			surveys during the summer season when considered separately.

Cross validation:

Marine-Only Training Data: Using only marine data to train model, how well does model predict onto shoreline survey data?

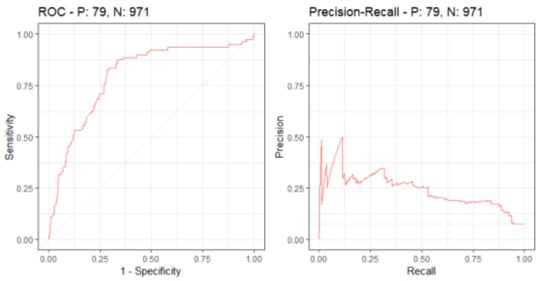

ROC: 0.79

PRC:0.23

Shoreline-Only Training Data: Using only shoreline data to train model, how well does model predict onto marine survey data? Restricted to marine points within 3 km of

shoreline.

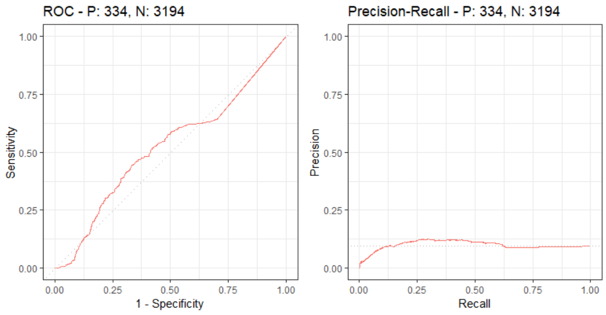

ROC: 0.52

PRC:0.10

Nonbreeding dataset

$logit(y_i) = \beta_0 + s(Longitude_i, Latitude_i, k=100) + s_2(NESTarea_i, by=SurveyType_i) + s(PSL_i, by=SurveyType_i) + s(STREAMdist_i, by=SurveyType_i) + s(SHOREdist_i) + s(Tidal_i, by=SurveyType_i) + s(NPGO_i) + SurveyType_i + Inlets_i + u(SurveyID_i) + v(Year_i) + offset(log(Effort_i))$

Model with Interactive Terms for Survey Type:

$logit(y_i) = \beta_0 + s(Longitude_i, Latitude_i, k=100) + s(NESTarea_i, by=SurveyType_i) + s(NESTcohweight_i) + s(PSL_i, by=SurveyType_i) + s(STREAMdist_i, by=SurveyType_i) + s(SHOREdist_i) + s(Tidal_i, by=SurveyType_i) + s(NPGO_i) + SurveyType_i + Inlets_i + u(SurveyID_i) + v(Year_i) + offset(log(Effort_i))$

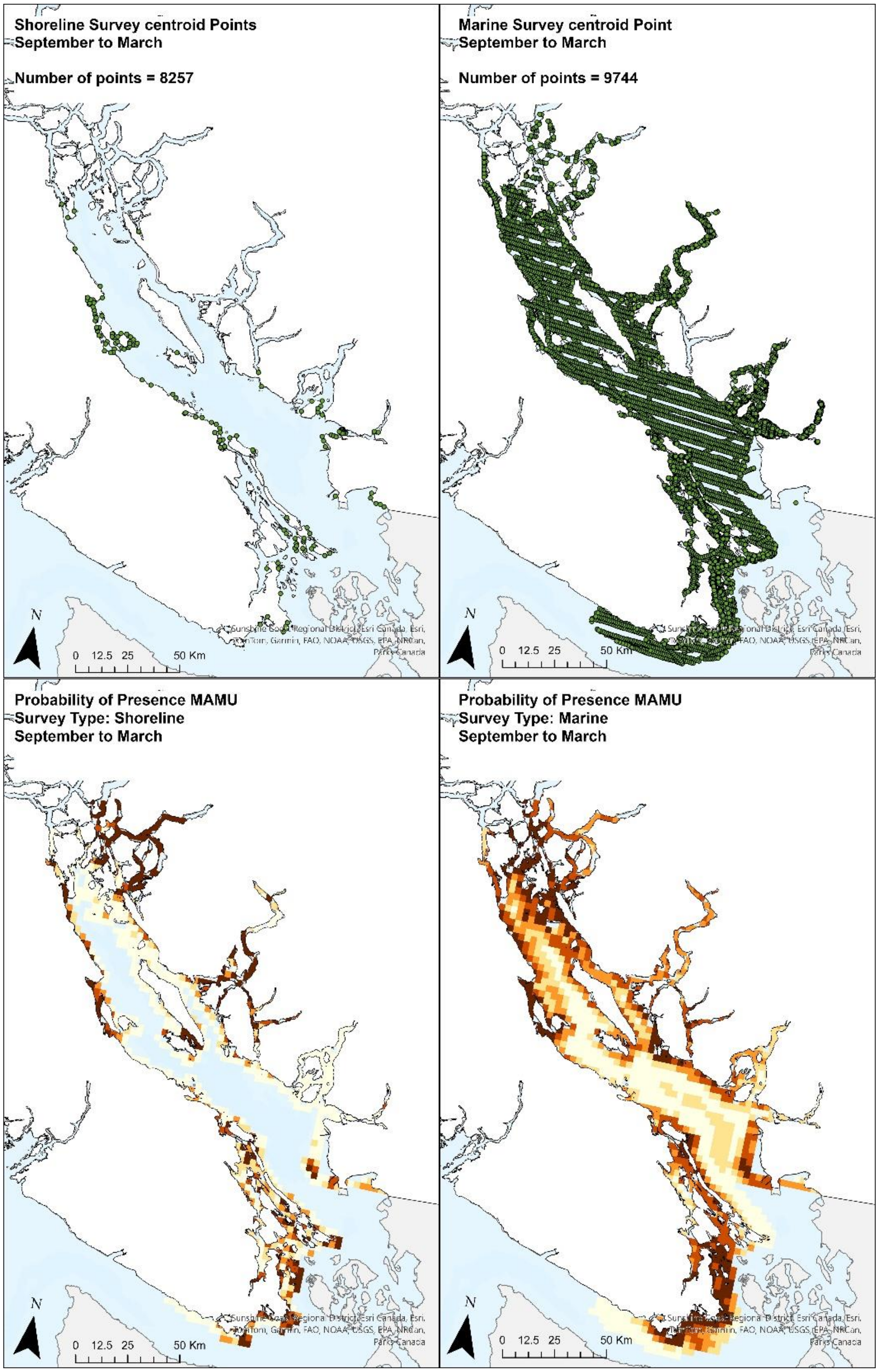

Fig C: Raw points and subsequent predictive Map using interactive Model with Survey Type Set to Shoreline Surveys (left) or Marine surveys (Right) during the nonbreeding season (Sept to March): Predictions are restricted to within 3 km of the shoreline for the shoreline survey prediction map.

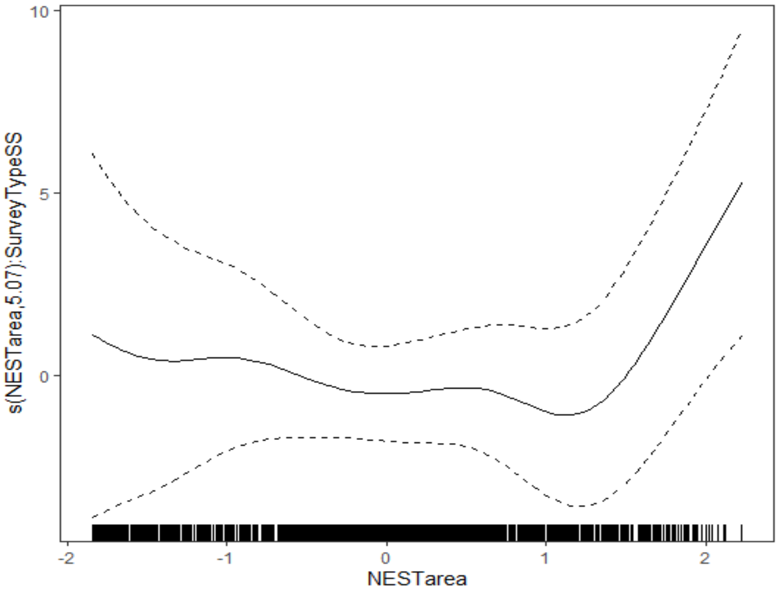

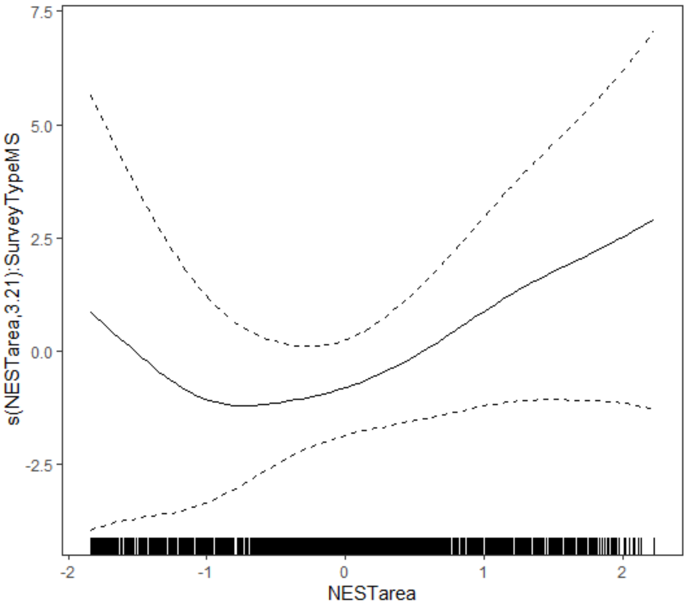

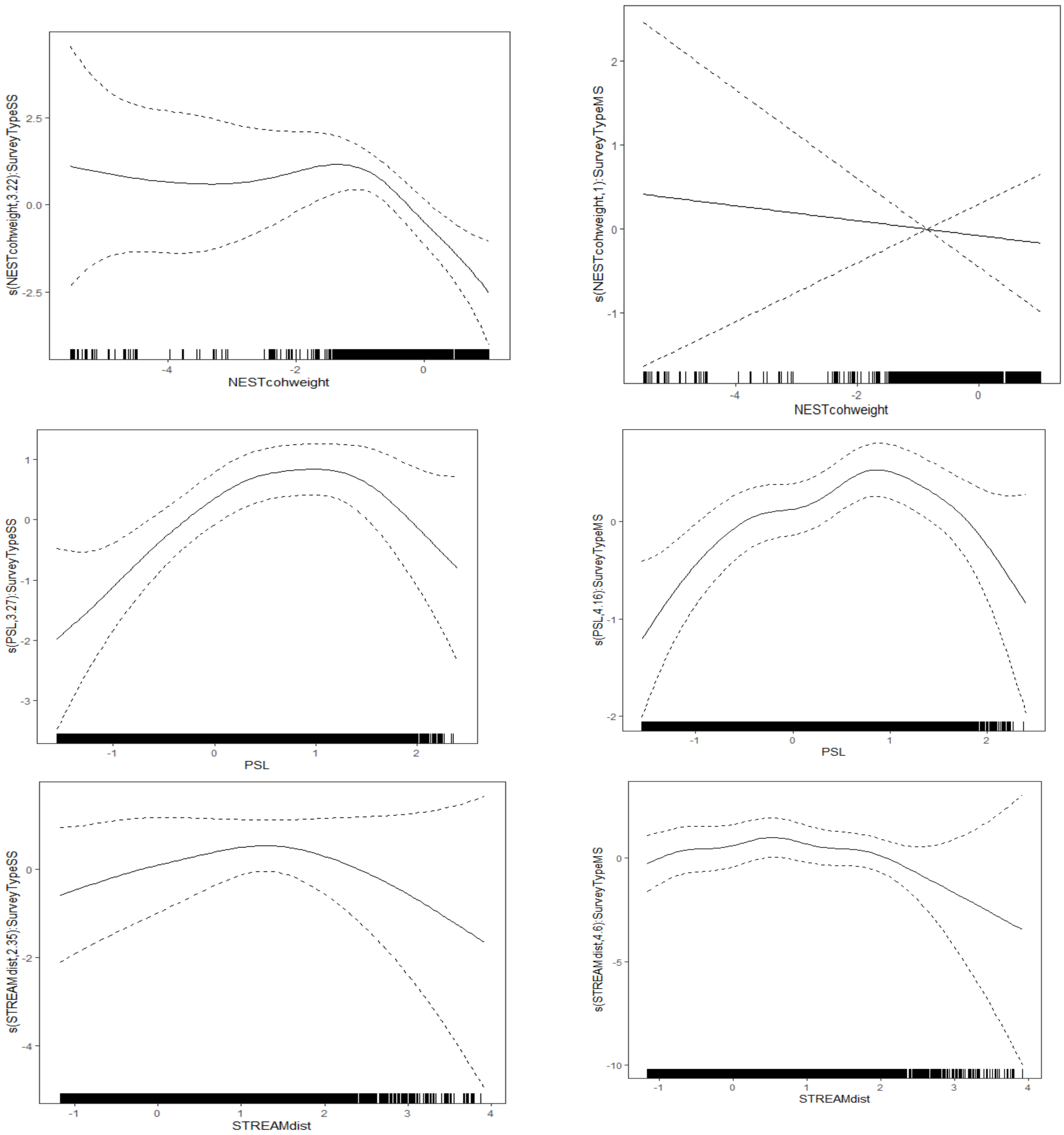

s(NESTcohweight,3.22):SurveyTypeSS
2.5
0.0
-2.5
-4
-2
0
NESTcohweight
s(NESTcohweight,1):SurveyTypeMS
2
1
0
-1
-4
-2
0
NESTcohweight
s(PSL,3.27):SurveyTypeSS
1
0
-1
-2
-3
-1
0
1
2
PSL
s(PSL,4.16):SurveyTypeMS
0
-1
-2
-1
0
1
2
PSL
s(STREAMdist,2.35):SurveyTypeSS
0
-2
-4
-1
0
1
2
3
4
STREAMdist
s(STREAMdist,4.6):SurveyTypeMS
0
-5
-10
-1
0
1
2
3
4
STREAMdist

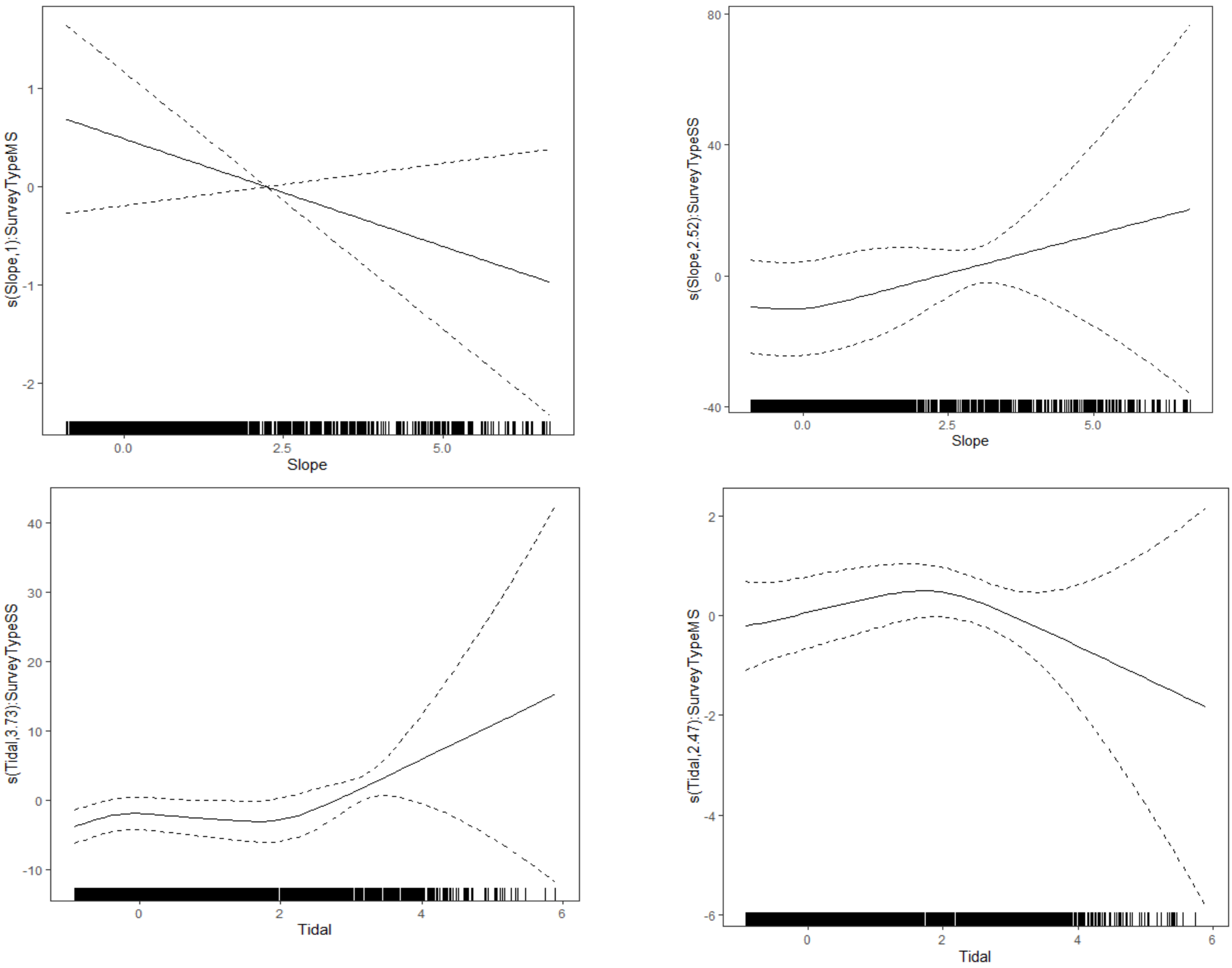

s(Slope,1):SurveyTypeMS
Slope
s(Slope,2.52):SurveyTypeSS
Slope
s(Tidal,3.73):SurveyTypeSS
Tidal
s(Tidal,2.47):SurveyTypeMS
Tidal

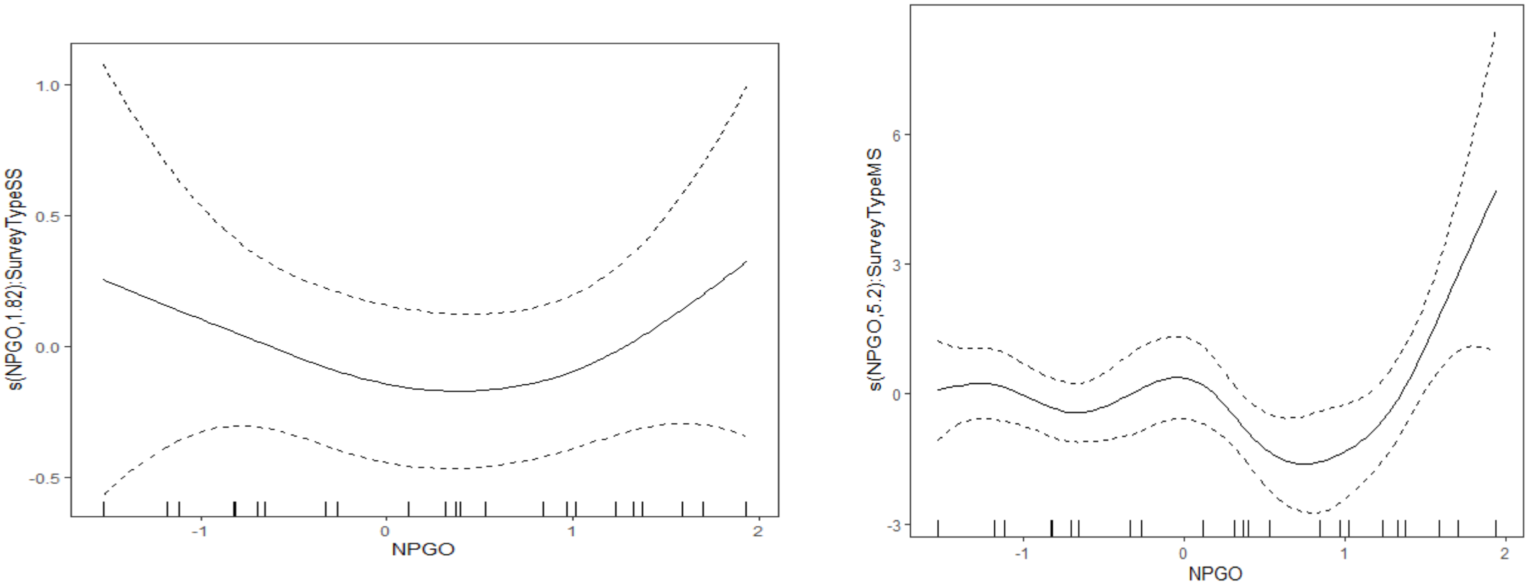

Fig D: Partial plot comparisons of marine and shoreline GAM outputs for the nonbreeding season dataset.

Table B: Summary of significant covariates for marbled murrelet presence during the nonbreeding season, based on Generalized Additive Model (GAM) outputs. The table presents the significance levels and p-values for each covariate, comparing their effects in marine surveys (MS) and shoreline surveys (SS).

Covariate	Marine (MS)	Shoreline (SS)	Explanation
NESTarea	Not Significant	Significant (p=0.001340)	NESTarea is significant only in shoreline surveys during the nonbreeding season when considered separately.
NESTcohweight	Not Significant	Significant (p=0.005064)	NESTcohweight is significant only in shoreline surveys during the nonbreeding season when considered separately.
PSL	Significant (p=0.002248)	Significant (p=0.001488)	PSL is significant in both environments during the nonbreeding season when considered separately.

Slope	Not Significant	Significant (p=5.96e-05)	Slope is significant only in shoreline surveys during the nonbreeding season when considered separately.
Tidal	Not Significant	Significant (p=0.003711)	Tidal conditions are significant only in shoreline surveys during the nonbreeding season when considered separately.

Cross validation:

Marine-Only Training Data: Using only marine data to train model, how well does model predict onto shoreline survey data?

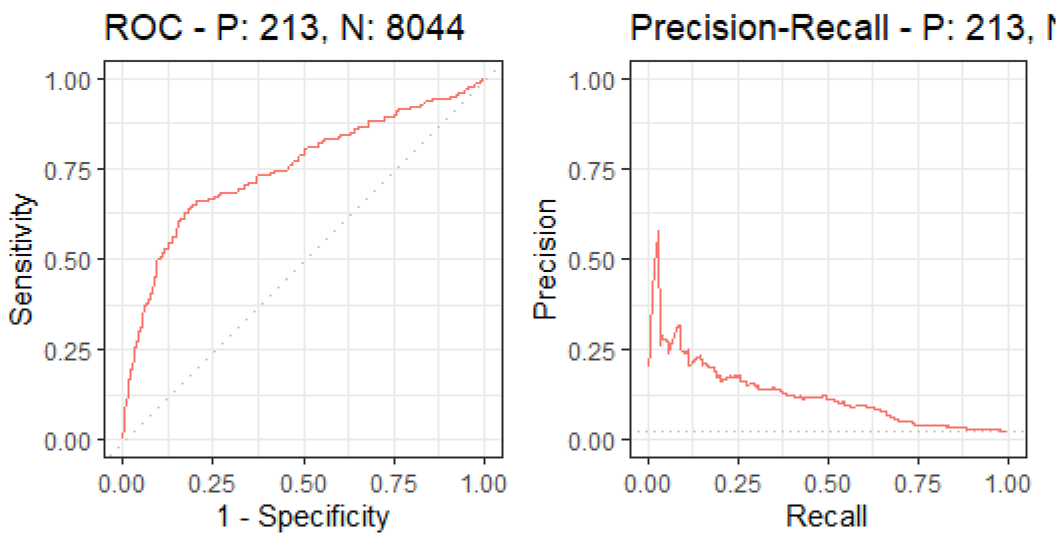

ROC: 0.74

PRC:0.12

Shoreline-Only Training Data: Using only shoreline data to train model, how well does model predict onto marine survey data? Restricted to marine points within 3 km of

shoreline.

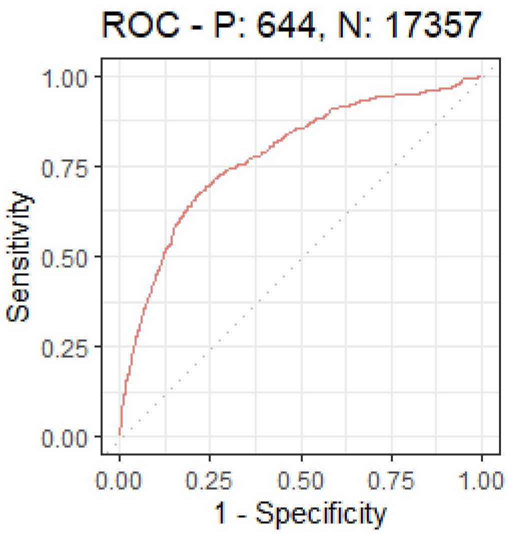

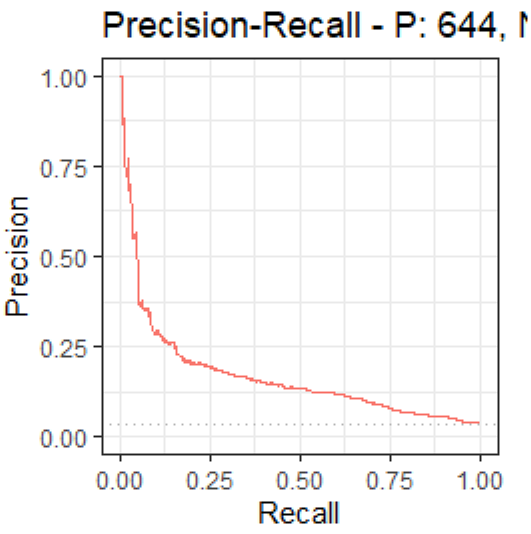

ROC: 0.78

PRC:0.17
