# Supplementary material for: Predicting marine habitat for marbled murrelets during breeding and nonbreeding seasons in the Salish Sea, British Columbia, Canada
Source: PLoS One. 2025 Jan 16;20(1):e0316946. doi: 10.1371/journal.pone.0316946 (PMC11737741; doi:10.1371/journal.pone.0316946)
Supplement: S2 File — (PDF) [file pone.0316946.s011.pdf]

Title: Predicting marine habitat for marbled murrelets during breeding and nonbreeding seasons in the Salish Sea, British Columbia, Canada

Sonya A. Pastran [1,*], Patrick D. O'Hara[2,3], Caroline H. Fox[4], Mark C. Drever[5], Ross Vennesland[6], Douglas F. Bertram[1]

S2 File Methods Detailed:

Spatial layers of suitable intertidal and subtidal habitat for Pacific sand lance were obtained from predictive models based on a maxent framework [22,23]. The original habitat layers had resolutions of 20 and 50 meters, respectively, and were developed to predict the likelihood of Pacific sand lance presence. These layers were combined into a single raster in ArcGIS Pro 3.1.0 using the merge tool, and the resolution was aggregated to a uniform 50 meters. To extract the Pacific sand lance habitat suitability information, the average probability of presence value within a 3 km grid radius of the observation points was extracted using the raster package in R 4.2.1. Bathymetry data with a spatial resolution of 100 m were downloaded from the British Columbia Marine Conservation Analysis (BCMCA) webpage [44]. The slope was derived from the bathymetry layer using the Spatial Analyst Slope tool in ArcGIS Pro 3.1.0. Average values within a 3 km radius of the survey centroids were extracted using the raster package in R 4.2.1.

Tidal current information was extracted from modeled values generated by Foreman et al. (2008) [45], with all data downloaded from BCMCA. The tidal speed values used were the root mean square (RMS) tidal speeds over modelled tidal cycles. Average tidal currents within a 3 km radius were extracted.

To determine the distance between the survey centroid and shorelines as well as streams, the *Near* tool in ArcGIS Pro 3.1.0 was utilized. For stream information, the British Columbia Stream Atlas Network was accessed, specifically stream orders 3 and higher, which can be retrieved from the online British Columbia data catalogue [46]. We opted to specifically consider stream orders of 3 and higher, given

their greater influence on the overall water flow in river systems [46]. This decision aligns with the importance assigned to stream orders of 3 or above in a habitat modeling study for marbled murrelets conducted in northern British Columbia [13].

The North Pacific Gyre Oscillation (NPGO) is a mode of climate variability in the North Pacific Ocean that is driven by changes in salinity, wind patterns and productivity [43,47]. It is characterized by fluctuations in sea surface height and temperature, as well as changes in ocean currents and nutrient availability. Data were available online [43,47], and we downloaded NPGO data spanning our study years (2000-2022). We binned the data by averaging the NPGO values by murrelet breeding and nonbreeding season and year.

A murrelet nesting habitat suitability layer was originally developed in 2010 to predict the amount and distribution of potential nesting habitat for murrelets on the coast of British Columbia [36]. The suitability layer is an amalgamation of outputs from several models that consider factors essential for murrelet nesting habitat, such as elevation, distance from the coast, and forest cover characteristics like tree height, tree age, and availability of platforms. Model outputs have been periodically updated through time to improve mapping accuracy and account for changes in land cover, with the latest update having occurred in 2018 [48]. Previous research has shown that the amount of available nesting habitat, its cohesion, and distance to suitable areas are related to murrelets' marine distribution and abundance [8,11,14]. To explore these relationships, we calculated four covariates from the nesting suitability model. Using the *landscapemetrics* package in R 4.2.1, we first calculated the total area of nesting habitat within an 80 km radius from marine points. We also calculated a cohesion metric within the same radius [49]. The cohesion metric considers the proximity and connectivity of these elements to evaluate the overall connectedness or fragmentation of the landscape. The calculation of cohesion involves assessing the degree to which patches are spatially linked. For our analysis, cohesion was characterized by the connectedness of suitable nesting habitat. Next, we calculated the same two

metrics but used inverse distance summed weights equal to 1, to capture how distance played a role in habitat selection. We used a weight of 0.7 for distances from 0 to 30 km from the marine point, a weight of 0.2 for distances from 30 to 50 km, and a weight of 0.1 for distances from 50 to 80 km. These distances were chosen based on decreasing importance to murrelets [7,8]. We selected the weights based on our own tests, as there is no existing literature for selecting inverse distance weighting values in this context. We tested a series of different weighted values, loosely based on the assumption that closer distances should have a higher weight compared to those farther away. After running several models with various weights, we chose the combination that had the strongest influence on the model.
